# Circulating tumor cell characterization of lung cancer brain metastases in the cerebrospinal fluid through single‐cell transcriptome analysis

**DOI:** 10.1002/ctm2.246

**Published:** 2020-12-15

**Authors:** Haoyu Ruan, Yihang Zhou, Jie Shen, Yue Zhai, Ying Xu, Linyu Pi, Ruofan Huang, Kun Chen, Xiangyu Li, Weizhe Ma, Zhiyuan Wu, Xuan Deng, Xu Wang, Chao Zhang, Ming Guan

**Affiliations:** ^1^ Department of Clinical Laboratory Huashan Hospital Fudan University Shanghai China; ^2^ Translational Medical Center for Stem Cell Therapy and Institute for Regenerative Medicine Shanghai East Hospital Shanghai Key Laboratory of Signaling and Disease Research School of Life Sciences and Technology Tongji University Shanghai China; ^3^ Department of Pathobiology Auburn University Auburn Alabama; ^4^ 10K Genomics Technology Co., Ltd. Shanghai China; ^5^ Department of Oncology Huashan Hospital Fudan University Shanghai China; ^6^ Department of Clinical Laboratory Huashan Hospital North Fudan University Shanghai China; ^7^ Central Laboratory Huashan Hospital Fudan University Shanghai China; ^8^ HudsonAlpha Institute for Biotechnology Huntsville Alabama; ^9^ Alabama Agricultural Experiment Station Auburn University Auburn Alabama

**Keywords:** cerebrospinal fluid, circulating tumor cell, leptomeningeal metastases, lung adenocarcinoma, single‐cell RNA sequencing

## Abstract

**Background:**

Brain metastases explain the majority of mortality associated with lung cancer, which is the leading cause of cancer death. Cytology analysis of the cerebrospinal fluid (CSF) remains the diagnostic gold standard, however, the circulating tumor cells (CTCs) in CSF (CSF‐CTCs) are not well defined at the molecular and transcriptome levels.

**Methods:**

We established an effective CSF‐CTCs collection procedure and isolated individual CSF cells from five lung adenocarcinoma leptomeningeal metastases (LUAD‐LM) patients and three controls. Three thousand seven hundred ninety‐two single‐cell transcriptomes were sequenced, and single‐cell RNA sequencing (scRNA‐seq) gene expression analysis was used to perform a comprehensive characterization of CSF cells.

**Results:**

Through clustering and expression analysis, we defined CSF‐CTCs at the transcriptome level based on epithelial markers, proliferation markers, and genes with lung origin. The metastatic‐CTC signature genes are enriched for metabolic pathway and cell adhesion molecule categories, which are crucial for the survival and metastases of tumor cells. We discovered substantial heterogeneity in patient CSF‐CTCs. We quantified the degree of heterogeneity and found significantly greater among‐patient heterogeneity compared to among‐cell heterogeneity within a patient. This observation could be explained by spatial heterogeneity of metastatic sites, cell‐cycle gene, and cancer‐testis antigen (CTA) expression profiles as well as the proportion of CTCs displaying mesenchymal and cancer stem cell properties. In addition, our CSF‐CTCs transcriptome profiling allowed us to determine the biomarkers during the progression of an LM patient with cancer of unknown primary site (CUP).

**Conclusions:**

Our results will provide candidate genes for an RNA‐based digital detection of CSF‐CTCs from LUAD‐LM and CUP‐LM cases, and shed light on the therapy and mechanism of LUAD‐LM.

## Graphical abstract

Our study firstly investigated CSF‐CTCs characteristics of five LUAD‐LM patients and one CUP‐LM patient.

CSF‐CTCs enriched metabolism and cell‐adhesion pathways.

CSF‐CTCs exhibited heterogeneity manifested by cell cycle, mesenchymal and cancer stem cell, and cancer‐testis antigen properties

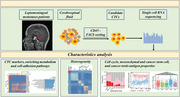

**Abbreviations**
BMs: brain metastases
CNS: central nervous system
CSC: cancer stem cell
CSF: cerebrospinal fluid
CTA: cancer‐testis antigen
CTCs: circulating tumor cells
CUP: cancer of unknown primary site
DEGs: differentially expressed genes
ECM: extracellular matrix
EMT: epithelial‐to‐mesenchymal transition
GSEA: gene set enrichment analysis
LMs: leptomeningeal metastases
LUAD: lung adenocarcinoma
MRI: magnetic resonance imaging
NSCLC: non‐small cell lung cancer
PDX: patient‐derived xenograft
scRNA‐seq: single‐cell RNA sequencing
t‐SNE: t‐distribution stochastic neighbor embedding

## 1 BACKGROUND

Lung cancer is the second common cancer type in both men and women.^1^ Non‐small cell lung cancer (NSCLC) is the main type of lung cancer, accounting for 85% of lung malignancies with a 5‐year survival rate less than 15%.^2^ Histologically, NSCLC is further classified into three subtypes: lung adenocarcinoma (LUAD), squamous‐cell carcinoma and large cell carcinoma, among which LUAD is the most common histological subtype.^3^, ^4^ Brain is the most common metastatic site of NSCLC, and the incidence of brain metastases (BMs) ranges from 22% to 54%, occurring at different stages of tumorigenesis but especially in advanced patients.^5^ Of all cancer patients with BMs, lung cancer is the primary tumor in 40‐50% cases, which is the highest among all cancer types and equals all other primary cancer types combined.^6^, ^7^ Leptomeningeal metastases (LMs) result from dissemination of cancer cells to both the leptomeninges (pia and arachnoid) and cerebrospinal fluid (CSF) compartment.^8^ Many chemotherapies for cancer have relatively poor central nervous system (CNS) penetration, allowing tumor cells to survive in the CNS and develop into LMs. LMs occur in 3‐5% of patients with advanced NSCLC and are most frequent in the LUAD subtype (LUAD‐LM).^9^ Although recent treatment advances including intrathecal chemotherapy, molecularly targeted therapy, and immunotherapy can prolong survival of LUAD‐LM patients to some extent, the outcomes of LUAD‐LM remain poor.^10^

The diagnosis and monitoring of NSCLC‐LMs are mainly based on medical history, clinical symptoms, imaging and CSF examinations. Gadolinium‐enhanced magnetic resonance imaging (MRI) of the brain and spine is the best imaging technique for LMs detection in solid tumors, with sensitivity of 70‐85% and specificity of 75‐90%.^10^ CSF sampling through lumbar puncture is of great importance in the diagnosis of NSCLC‐LM and a positive CSF cytology result remains the diagnostic gold standard.^10^ The CellSearch technique, which utilizes immunomagnetic selection, identification, and quantification of CSF‐circulating tumor cells (CTCs), is more sensitive than conventional cytology and MRI for the diagnosis of LMs.^11^ However, the CSF‐CTCs have great heterogeneity, epithelial cells in the epithelial‐to‐mesenchymal transition (EMT) process contribute to the false negatives of CellSearch capture.^12^ Recently, assessment of an RNA‐based molecular signature using a droplet digital polymerase chain reaction (PCR) assay constituted a greatly sensitive and specific CTC readout, enabling high‐throughput clinical applications and allowing for early diagnosis and metastasis prediction of cancer to improve prognosis of patients.^13^, ^14^ Therefore, it is meaningful to establish an RNA‐based digital detection of CSF‐CTCs to help diagnose LUAD‐LM. However, the transcriptome characteristics of CSF‐CTCs from LUAD‐LM patients are still unknown and deserve to be studied.

CSF‐CTCs are relatively rare in patient CSF samples, and ≥1 CSF‐CTC/mL was defined as a cutoff for diagnosis.^15^ The traditional profiling technologies that measure tumor cells in bulk have been confounded by the presence of normal lymphocytes and cannot capture gene expression heterogeneity among tumor cells. Therefore, we investigated the transcriptome characteristics of CSF‐CTCs by Smart‐seq2 single‐cell RNA sequencing (scRNA‐seq).^16^ We enrolled five LUAD‐LM patients and investigated the transcriptional profiles for more than one thousand CSF‐CTCs. By analyzing the transcriptome characteristics of CSF‐CTCs from LMs patients at the single‐cell level, the intra‐tumoral and inter‐tumoral heterogeneity of CSF‐CTCs can be revealed for the first time. In addition, the discovery of characteristic genes of CSF‐CTCs could be utilized to combine an RNA‐based molecular signature for further clinical diagnosis, as well as to facilitate potential breakthroughs in tackling the clinical challenge of LMs.

## 2 MATERIALS AND METHODS

### 2.1 Patients’ information and sample collection

All human sample materials used in this research were collected at Huashan Hospital, Fudan University. The consent forms and the proposed studies were approved by Institutional Review Board of Huashan Hospital (HIRB, KY2019‐002). Patients diagnosed as LUAD‐LM (P1, P2, P4, P6, and P7) or CUP‐LM (P8) without any other cancers were included in the study. Three CSF samples (N1‐N3) were collected from patients who had pulmonary cryptococcal infection without CNS symptoms, and screened for potential CNS infections. The examination results showed the three CSF samples (N1‐N3) were normal without cryptococcal infection. Clinical information of patients is listed in Table S1.

### 2.2 Cell sorting and single‐cell preparation

**FIGURE 1:**
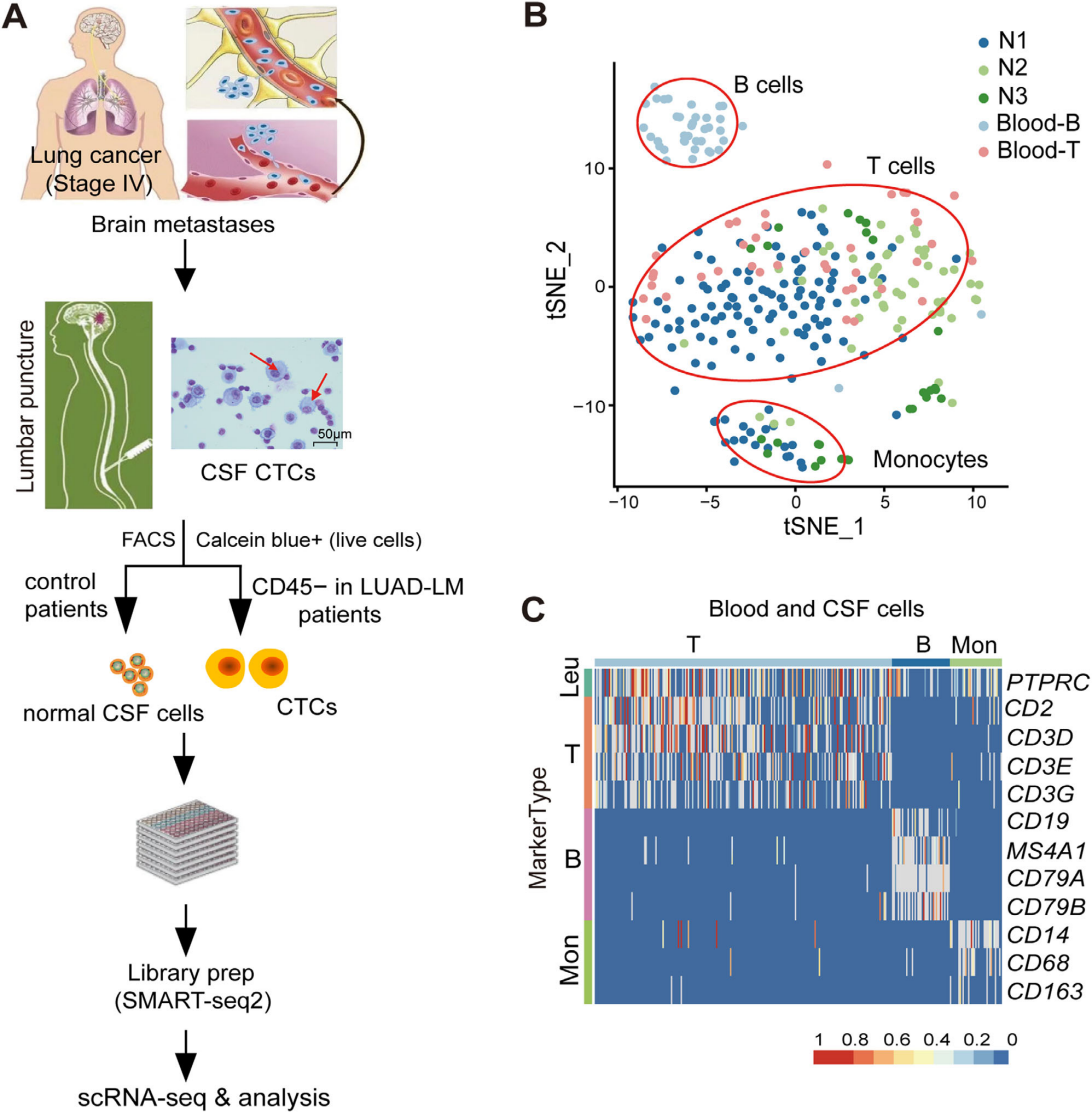
Isolation of cerebrospinal fluid (CSF) circulating tumor cells (CTCs) and characterization of normal CSF cell composition at the single‐cell level. A, CSF‐CTCs isolation workflow. CSF samples were collected from lung adenocarcinoma leptomeningeal metastases (LUAD‐LM) patients. Under CSF cytology (magnification 400×; Wright's stain; scale bar represents 50 μm), CTCs showing a larger cell diameter (red arrows) than lymphocytes. Individual live cells were sorted by fluorescence‐activated cell sorting (FACS) for single cell RNA sequencing (scRNA‐seq) following the Smart‐seq2 protocol. Illustration of LUAD‐BM is modified from Figure 1 in Ghaffari et al. B, t‐distributed stochastic neighbor embedding (t‐SNE) plot of cells from control CSF samples (N1, N2, and N3) and blood T/B cells, showing the cell identity and gene expression correlations. C, High‐resolution heatmap showing expression of selected marker genes in blood and CSF cells Abbreviations: Leu, leukocytes; Mon, monocytes.

Antibodies (CD45, catalogue number Cat: 560973; CD3, Cat: 561806; CD19, Cat: 564456; BD Biosciences) and labeling dye for live cells (Calcein Blue AM, Life Technologies, CA, Cat: C34853) were used per manufacturer recommendations. Pathological CSF samples were diagnosed by cytology, and CSF‐CTCs in 3 mL leftover CSF per patient sample (Table S1) with a larger cell diameter than lymphocytes were selected by fluorescence‐activated cell sorting (FACS; Calcein Blue AM+, CD45−; Figure 1A; Figure S1) for scRNA‐seq following the Smart‐seq2 protocol. For control purposes, live cells (Calcein Blue AM+) from 3 mL leftover CSF per normal sample (Normal, N1‐N3; Figure 1A; Table S1) were processed using the same pipeline except for the cell selection. Among all of the CSF samples, 3792 single cells were selected for sequencing (N: 624 cells; P: 3,168 cells; Table S1). In addition, 168 blood T cells (Calcein Blue AM+, CD45+, CD3+) and 168 blood B cells (Calcein Blue AM+, CD45+, CD19+) were also sorted for sequencing (Table S1). Targeted cells were sorted into pre‐prepared 96‐well plates by FACS. Single‐cell lysates were sealed, vortexed, centrifuged, placed on dry ice and transferred immediately for storage at −80°C.

### 2.3 Smart‐seq2 library construction and sequencing

Libraries for isolated single cells were generated by the Smart‐seq2 method^16^ with the following modifications: RNA was reverse transcribed with Maxima H Minus Reverse Transcriptase (Thermo Fisher Scientific, MA, Cat: 00724792), and whole transcriptome was amplified using KAPA HiFi Hot Start Ready Mix (KAPA Biosystems, MA, Cat: KE2502). cDNA library was purified using Agencourt XP DNA beads (Beckman Coulter, CA, Cat: A63852) and quantified with a high sensitivity dsDNA Quant Kit (Life Technologies, CA, Cat: Q32854). It is worth mentioning that full length cDNA libraries were tagmented, and then only 3′ end sequence (500‐1000 bp) was amplified and enriched for sequencing on an Illumina HiSeqX machine, which is different from the traditional Smart‐seq2 method of full tagmented‐libraries sequencing.

### 2.4 scRNA‐seq expression analysis

The Illumina sequencing data were demultiplexed based on sample barcodes. Adapter sequences, poly T, and residue barcodes were trimmed using custom scripts. After removing UMIs and low‐quality bases, the filtered reads were aligned to the human reference genome (hg19) by STAR,^17^ and BAM files were prepared by SAMtools.^18^ Gene expression counts were obtained by FeatureCounts.^19^

Genes expressed in less than 10 cells were filtered out from the gene expression matrix of CSF samples. Individual cells with fewer than 600 covered genes and over 20% mitochondrial reads were filtered out, and 1986 single cells remained (401 immune cells and 1585 CTCs) for subsequent analysis using the Seurat 3.0 software package^20^ (Table 1). The mean number of genes detected per cell was 830 for immune cells and 1870 for tumor cells, respectively.

**TABLE 1:** Summary of cell type identity of cells in patient CSF samples

		Cell selection						
Patient ID	Diagnostics	Calcein blue AM	CD45	Number of sequenced cells	Number of QC filtered cells	T cells	Monocytes	CTCs
P1‐1	LUAD‐LM	+	−	168	99	0	0	99
P1‐2	LUAD‐LM	+	−	360	280	0	0	280
P2	LUAD‐LM	+	−	192	130	0	0	130
P4	LUAD‐LM	+	−	288	157	0	15	142
P6	LUAD‐LM	+	−	480	281	0	0	281
P7	LUAD‐LM	+	−	288	205	0	0	205
P3	LUAD‐LM	+	N/A	96	50	0	50	0
P8‐1	CUP‐LM	+	−	480	152	0	0	152
P8‐2	CUP‐LM	+	−	816	343	28	19	296
**Total**				**3,168**	**1,697**	**28**	**84**	**1,585**

Abbreviations: Calcein Blue AM, labeling dye for live cells selection; CD45, protein tyrosine phosphatase receptor type C, marker for leukocytes; CUP‐LM, leptomeningeal metastases of cancer of unknown primary site; LUAD‐LM, lung adenocarcinoma leptomeningeal metastases; N/A: Not applicable; QC, quality control.

When we analyzed the transcriptome characteristics of CTCs, we selected tumor cells with more than or equal to1000 covered genes, and 1360 CTCs retained (340 from P1, 122 from P2, 127 from P4, 206 from P6, 172 from P7, 393 from P8) for analysis. The mean number of genes detected per CSF‐CTCs was 2070.

### 2.5 Clustering and marker expression analysis for cell type identification

Cells were clustered by non‐supervised t‐distribution stochastic neighbor embedding (t‐SNE) dimensionality reduction^21^ based on their gene expression counts. The cells were separated into groups with indication of cryptic inner‐group connection. The cluster‐specific marker genes were identified by the FindAllMarkers function in Seurat 3.0. Single‐cell RNA‐seq data of two human LUAD patient‐derived xenograft (PDX) samples (LC‐PT‐45, PT45; LC‐MBT‐15, MBT15) and a human NSCLC cell line (H358 cell line) were obtained from NCBI Sequence Read Archive with accession number GSE69405.^22^ These data were filtered using the same pipeline as CSF‐CTCs, and only cells with more than or equal to 1000 genes were included for analysis.

To infer the cell type identity, Seurat 3.0 was used to generate expression heatmaps of selected gene markers of known cell types, including T cells (*CD2*, *CD3D*, *CD3E*, and *CD3G*), B cells (*CD19*, *MS4A1*, *CD79A*, and *CD79B*), monocytes (*CD14*, *CD68*, and *CD163*), lung cells (*SFTPA1*, *SFTPA2*, *SFTPB*, and *NAPSA*), epithelial cells (*EPCAM*, *CDH1*, *KRT7*, *KRT8*, *KRT18*, and *MUC1*), and proliferation cells (*CCND1* and *TOP2A*). The ImmuneScore was computed based on Estimation of STromal and Immune cells in MAlignant Tumors using Expression data (ESTIMATE) R package.^23^

### 2.6 Differentially expressed genes and pathway enrichment analysis

Significantly differentially expressed genes (DEGs) between samples were detected by DESeq2^24^ using normalized gene expression counts, at an adjusted *P*‐value cutoff of .05 and a fold‐change cutoff of 2. Gene set enrichment analysis (GSEA) was used for functional enrichment analysis of Kyoto Encyclopedia of Genes and Genomes pathways.^25^

### 2.7 Cell cycle analysis

Cell cycle assignment was performed in R version 3.6.0 using the CellCycleScoring function included in Seurat 3.0 package. Cycling cells are defined as cells with either G1/S.Score or G2/M.Score greater than 0.2. The criterion of intermediate cells is 0 < G1/S.Score or G2/M.Score ≤ 0.2. The rest cells are defined as non‐cycling cells.

### 2.8 Statistical analysis

All other *P* values except for DEGs analysis were obtained using Wilcoxon Rank‐Sum test, and *P* < .05 was considered significant.

## 3 RESULTS

### 3.1 Cell composition of CSF at the single‐cell transcriptome level

To characterize the single‐cell transcriptomes and the composition of CSF cells under healthy conditions, we sequenced the single‐cell transcriptomes of 624 cells from three normal CSF samples (N1‐N3) using Smart‐seq2 single‐cell RNA‐sEquation (scRNA‐seq) technology (Figure 1A; Table S1). In addition to CSF cells, blood T cells and B cells were sorted and sequenced to establish the cell type transcriptome profiles to help define the normal CSF cells composition. After quality filtering (Materials and Methods), 207 normal CSF cells, 41 B cells, and 41 T cells were clustered using t‐SNE method (Figure 1B). On average, 803 expressed genes were detected per cell. We identified three clusters corresponding to B cells, T cells, and monocytes defined by the expression patterns of leukocyte markers (Figure 1C). The Blood T and CSF T cells clustered together, indicating normal lymphocytes had similar expression profiles in different microenvironments. Normal CSF samples consisted of 80.3% T cells and 19.7% monocytes. No B cells were found in normal CSF samples (Table S2).

### 3.2 Identification and characterization of circulating tumor cells in the CSF of LUAD‐LM patients

**FIGURE 2:**
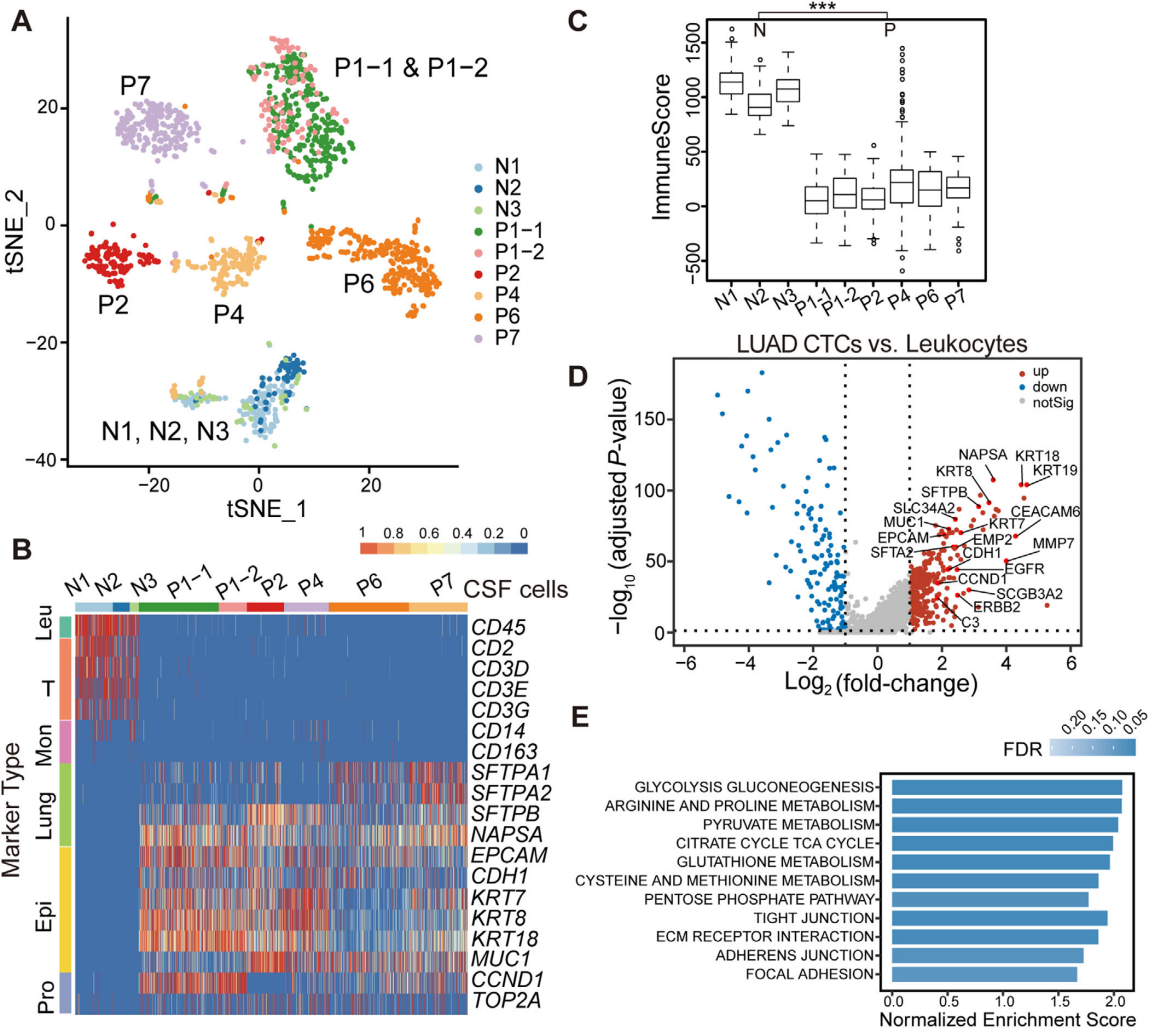
Characterization of LUAD‐LM (lung adenocarcinoma leptomeningeal metastases) CSF‐CTCs (cerebrospinal fluid circulating tumor cells) using single‐cell transcriptome analysis. A, t‐SNE (t‐distributed stochastic neighbor embedding) plot of gene expression clustering of three normal CSF samples (N1, N2, and N3) and five LUAD‐LM CSF samples (P1, P2, P4, P6, and P7). Clusters are assigned according to cell identity and gene expression correlations. B, Heatmap showing expression levels of selected marker genes in each sample (Leu, leukocytes; Mon, monocytes; Epi, epithelial; Pro, proliferation). C, Immune signature of CSF cells quantified by the ImmuneScore computed from the ESTIMATE algorithm, showing the significant difference between the normal (N) samples group (left) and the patient (P) samples group (Right) (***P < .001, Wilcoxon Rank‐Sum test). D, Volcano plot of differentially expressed genes profile between the CSF‐CTCs and leukocytes from control and patient CSF samples (adjusted *P*‐value < .05; fold‐change > 2). Gene names are labeled for selected genes upregulated in CSF‐CTCs. E, KEGG (Kyoto Encyclopedia of Genes and Genomes) pathways significantly enriched in LUAD CSF‐CTCs compared to leukocytes by gene set enrichment analysis (GSEA; FDR < 0.05)

We developed an effective and highly reproducible protocol for cell isolation from the CSF samples of LUAD‐LM patients, and six LM patients were enrolled in the scRNA‐seq study (Table S1). In total, 1776 candidate CTCs from five LUAD‐LM patients (P1, P2, P4, P6, and P7) were FACS sorted (CD45− and Calcein Blue AM+; Figure 1A; Figure S1) and sequenced, from which 1152 cells with at least 600 covered genes in their transcriptome were included in our analysis (Table 1), and these cells were clustered using the t‐SNE method along with the three normal CSF samples N1‐N3. The majority of patient CSF cells clustered according to the patient of origin, with the exception of 15 monocytes in P4 (Figure 2A). These patient CSF cells were candidate CTCs from the BMs. The clustering pattern was not driven by technical variability, because CSF samples collected from the same patient within a 2‐month time interval (P1‐1 and P1‐2) formed a single coherent cluster (Figure 2A), despite of independent cell sorting, library construction, and sequencing. There was no significant heterogeneity observed in mapping quality or gene coverage across patient samples (Figure S2A‐B; Table S3), suggesting that the clustering was not due to technical artifact.

The diameter of CSF‐CTC was larger than that of CSF normal cell (Figure 1A; Figure S1). To determine whether CTCs could be separated by morphology without CD45 selection, live cells were isolated from another LUAD‐LM patient (P3) merely based on cell morphology. Single‐cell transcriptome profiling revealed 100% of collected cells were monocytes (Figure S2C), indicating that the CD45 negative selection step is necessary for the successful isolation of CSF‐CTCs.

At the molecular level, we defined CSF‐CTCs as non‐immune cells with transcriptome signatures for LUAD markers, epithelial markers, and proliferation markers (Figure 2B; Figure S3). Based on these criteria, 100% of cells in P1, P2, P6, and P7 CSF samples and 90.4% (142/157) of P4 CSF cells were CTCs (Table 1). Compared to control CSF samples, patient CSF samples lacked expression of immune cell markers (Figure 2B; Figure S3) and had a significantly lower mean ImmuneScore^23^ (127 vs 1703, *P* < .001, Wilcoxon Rank‐Sum test; Figure 2C). Epithelial cell markers,^26^ including *EPCAM*, *CDH1*, *KRT7*, *KRT8*, *KRT18*, and *MUC1*, were expressed in CTCs, suggesting their epithelial origin (Figure 2B; Figure S3). LUAD markers surfactant protein A^27^ (*SFTPA1*, *SFTPA2)*, *SFTPB*,^28^ and *NAPSA* ^29^ were also expressed in most CTCs, indicating that they originated from primary lung cancer tumor cells (Figure 2B; Figure S3). CTCs also expressed common proliferation markers *CCND1* and *TOP2A* (Figure 2B; Figure S3).

### 3.3 Transcriptome signatures of CSF‐CTCs in LUAD‐LM patients

Two hundred ninety genes were significantly upregulated in patient CSF‐CTCs compared to normal CSF cells (adjusted *P*‐value *P*‐adj < .05 and log_2_fold‐change log_2_FC > 1; Figure 2D). Among these genes, *CEACAM6* (−log_10_ *P*‐adj = 67.71, log_2_FC = 4.27; Figure 2D) is a carcinoembryonic antigen cell‐adhesion molecule and a biomarker for mucinous adenocarcinoma. Overexpression of *CEACAM6* has been shown to associate with poor prognosis due to its roles in cellular invasiveness, resistance to anoikis and metastatic potential.^30^ *SCGB3A2*,^31^ another marker for pulmonary carcinoma, was also significantly upregulated (−log_10_ *P*‐adj = 29.96, log_2_FC = 2.83; Figure 2D). *SCGB3A2* is a member of the secretoglobin (SCGB) gene superfamily mainly found in bronchial epithelial cells. It is a growth factor during fetal lung development with anti‐inflammatory function in the lung.^32^, ^33^ Recently, C3 (−log_10_ *P*‐adj = 24.08, log_2_FC = 1.84; Figure 2D) from CSF cancer cells has been proved necessary for cancer growth within the leptomeningeal space.^34^ C3 activates the C3a receptor in the choroid plexus epithelium to disrupt the blood‐CSF barrier, allowing plasma amphiregulin and other mitogens to enter the CSF and promote cancer cell growth.^34^ As secreted proteins, the elevated expression of *CEACAM6*, *SCGB3A2*, and *C3* have great potentials in a developing CSF immunoassay for LUAD‐BM diagnosis.

Energy metabolism category and cell adhesion category were significantly enriched in CSF‐CTCs transcriptomes (FDR < 0.05; Figure 2E). The enhancement of glucose utilization involved in glycolysis gluconeogenesis pathway and citrate cycle TCA cycle pathway (FDR < 0.05; Figure 2E) in energy metabolism category is critical for the energy demand of brain.^35^ In addition, enhanced activation of the pentose phosphate pathway and glutathione metabolism pathway (FDR < 0.05; Figure 2E) can minimize oxidative stress, which is beneficial for metastatic cells to survive in the brain.^36^ The up‐regulated cell adhesion category consisted of tight junction pathway, extracellular matrix (ECM) receptor interaction pathway, and adhesion junction pathway (FDR < 0.05; Figure 2E), indicating that CSF‐CTCs possessed a higher adhesion strength, which is crucial for essential functions such as survival, proliferation, migration, and the ability to maneuver through capillary‐sized vessels to a new location.^37^, ^38^

In summary, CSF‐CTCs had unique gene expression profiles with full capacity of cancer‐ and metastasis‐related functions. The single‐cell transcriptome characteristics reassured that most of patient CSF cells were indeed CSF‐CTCs.

### 3.4 Spatial and gene expression heterogeneity of LUAD‐LM tumors

**FIGURE 3:**
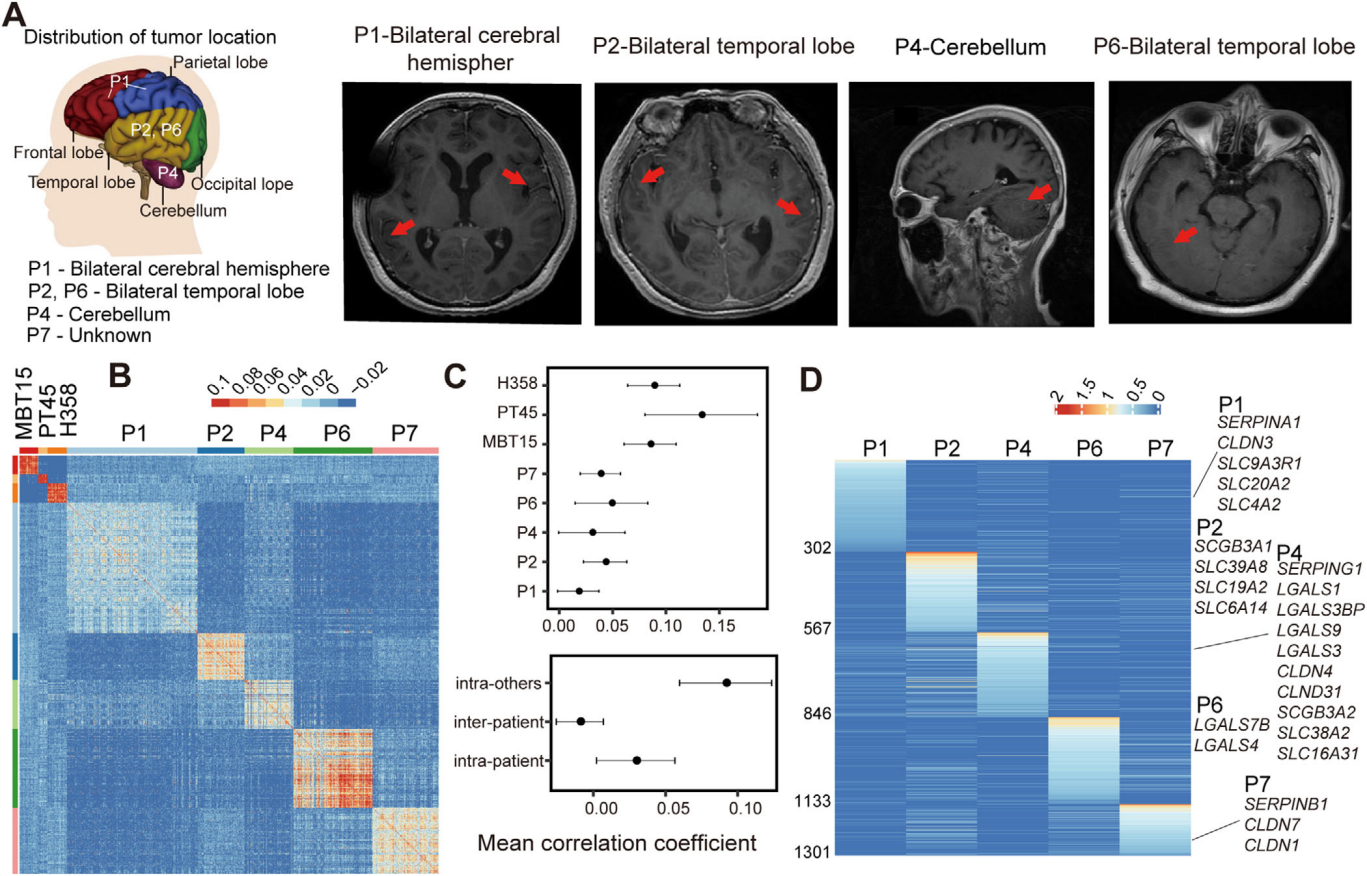
The heterogeneity of LUAD‐LM (lung adenocarcinoma leptomeningeal metastases) CSF‐CTCs (cerebrospinal fluid‐circulating tumor cells) among different patients and within individual patients. A, Locations of LMs in LUAD‐LM patients reveal spatial heterogeneity. Brain MRI (magnetic resonance imaging) diagnostic results of patients P1, P2, P4, and P6 are shown, demonstrating leptomeningeal enhancement (red arrows, Table S1). B, Heatmap showing pairwise correlations at the single‐cell transcriptome level for CSF‐CTCs of LUAD‐LM patients, human non‐small cell lung cancer cell line H358, and human lung adenocarcinoma patient‐derived xenograft (PDX) cells MBT15 and PT45. C, Top: degree of heterogeneity among cells measured by the mean correlation coefficient within individual samples. Bottom: heterogeneity analysis showing the mean correlation coefficient for CTCs within individual CSF samples (intra‐patient), among CSF samples (inter‐patient), and for cells in two individual PDX samples and H358 cell line (intra‐others). D, Heatmap of differentially expressed genes (*P*‐value < .05, fold‐change > 1.5) that are exclusively or preferentially expressed in one individual LUAD‐LM patient. The names of selected genes are labeled

LMs often occur at different brain locations, resulting in spatial heterogeneity of the metastatic tumors. We examined the five LUAD‐LM patients and found this is exactly the case (Figure 3A). To investigate gene expression heterogeneity at the single‐cell level, we quantified pairwise correlations between the expression profiles of 967 single‐CTC transcriptomes from the five LUAD‐LM samples (Figure 3B), and discovered significant heterogeneity between CSF cells both among different patients (inter‐tumor) and within individual patients (intra‐tumor; correlation coefficients ranging from −0.057 to 0.829). Inter‐tumoral heterogeneity was significantly greater than the intra‐tumoral heterogeneity (mean correlation coefficient −0.009 vs 0.029, *P*‐value < 2.2e‐16, Wilcoxon Rank‐Sum test; Figures 3B and 3C). To compare with primary tumors, we utilized single‐cell expression data from a human NSCLC cell line H358, and two human LUAD PDX samples MBT15 and PT45.^22^ The cell‐to‐cell correlations within individual primary tumor samples were significantly higher than those within individual patient CSF samples (mean correlation coefficient 0.092 vs 0.029, *P*‐value < 2.2e‐16, Wilcoxon Rank‐Sum test), indicating greater heterogeneity in clinical CSF samples (Figures 3B and 3C).

A total of 1300 DEGs were identified in individual CSF samples (*P*‐value < .05, fold‐change > 1.5; Figure 3D). Functionally, important gene families varying across individual patients included serpins (SERPIN), galectins (LGAL), claudins (CLDN), SCGB, and solute carrier family (SLC), which are important contributors to the patient‐specific signatures (Figure 3D).

### 3.5 The majority of the CSF‐CTCs are in the non‐cycling state in LUAD‐LM patients

**FIGURE 4:**
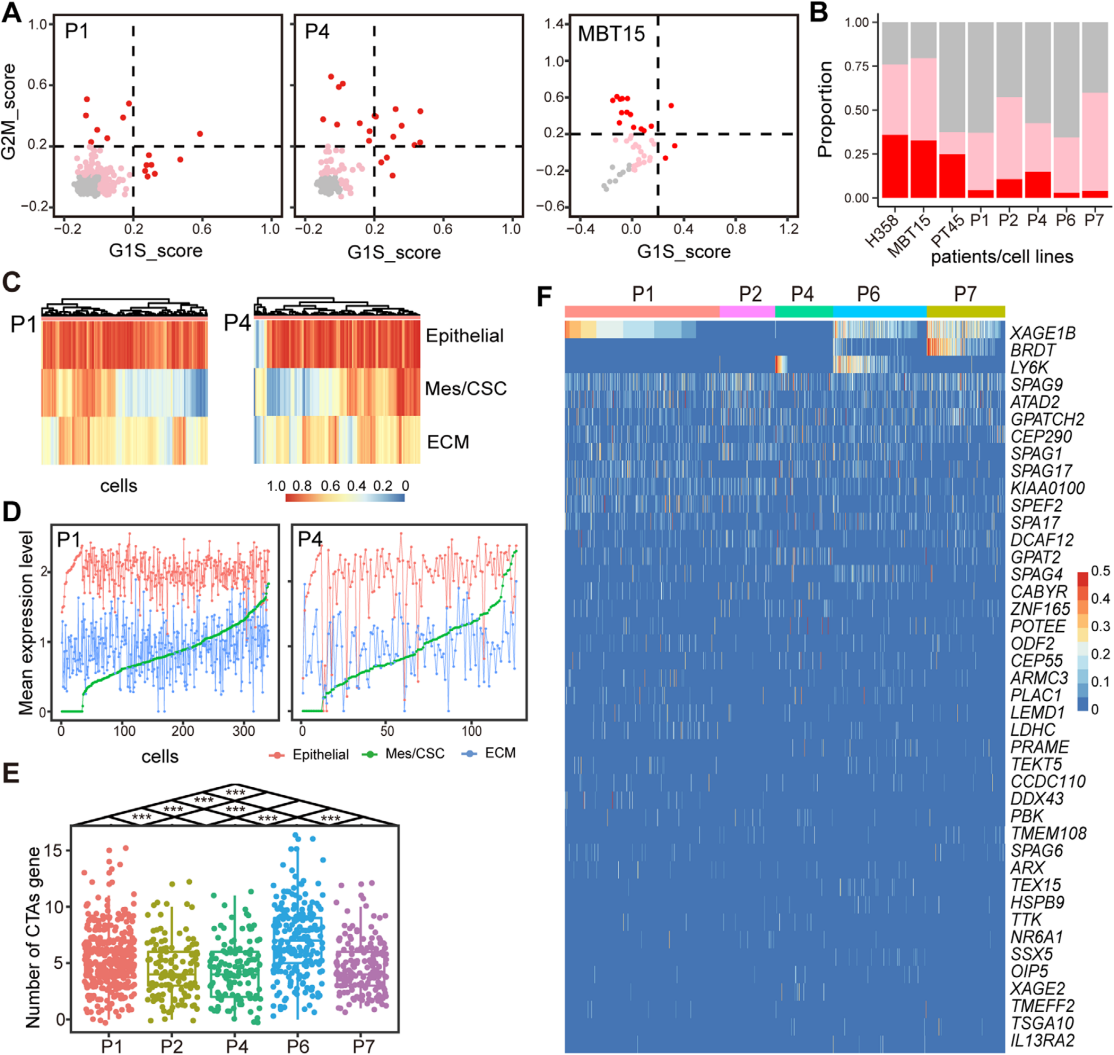
CSF‐CTCs (cerebrospinal fluid circulating tumor cells) gene expression profiles of cell cycle genes, cancer‐testis antigens (CTAs) and genes related to partial EMT (epithelial‐to‐mesenchymal transition). A, Cell cycle states of individual CSF‐CTCs (dots) are estimated based on relative expression of G1/S (*x*‐axis) and G2/M (*y*‐axis) genes in patients P1and P4, and human lung adenocarcinoma patient‐derived xenograft (PDX) cells MBT15. Cells are colored by inferred cell cycle states (cycling cells: score > 0.2, red; intermediate cells: 0 < score ≤ 0.2, pink; noncycling cells: score ≤ 0, gray). B, Summary of cell cycle state proportions (*y*‐axis) enriched in each sample (*x*‐axis). The color code is consistent with that of Figure 4A. C, Unsupervised clustering using the GSVA (gene set variation analysis) score of epithelial genes, mesenchymal/cancer stem cell (Mes/CSC) genes, and extracellular matrix (ECM) genes in P1 and P4 CSF‐CTCs. Epithelial genes: *CDH1*, *EPCAM*, *KRT18*, *KRT19*, *KRT7*, *KRT8*, and *MUC1*; Mes/CSC genes: *FN1* and *VIM*/*CD44*; ECM genes: *LAMA3*, *LAMA5*, *LAMB2*, *LAMC1*, *ITGA3*, *ITGB4*, and *CD47*. D, Line chart of average normalized expression levels (*y*‐axis) of epithelial genes (red), Mes/CSC genes (green) and ECM genes (blue) in P1 and P4 CSF‐CTCs. CSF‐CTCs are ranked by average normalized expression level of Mes/CSC genes (*x*‐axis). E, Boxplot of the number of CTAs (*y*‐axis) expressed in CSF‐CTCs (dots) from five patients (*x*‐axis), showing the difference between any two patients (***P < .001, Wilcoxon Rank‐Sum test). F, Heatmap of single‐cell expression profiles of CTAs in CSF‐CTCs from five patients

LUAD‐LM patients tend to have poor prognosis with residual tumor cells that disseminate rapidly in CSF within several months.^10^ We analyzed the cell‐cycle state of the CSF‐CTCs based on the single‐cell transcriptomes. On average, high‐cycling cells only accounted for 7.2% in LUAD‐LM patients (4% in P1, 11% in P2, 14% in P4, 3% in P6, and 4% in P7), which was much fewer than that observed in the H358 cell line (36%) and two PDX samples (33% in MBT15 and 25% in PT45) (Figures 4A and 4B; Figure S4A).

### 3.6 Cancer stemness and partial EMT in CSF‐CTCs

Cancer stem cells (CSCs) exhibit the properties of asymmetrical division, differentiation, and self‐renewal, as well as increased intrinsic resistance to therapy.^39^ The expression levels of lung CSC candidate biomarkers were investigated, including *PROM1*(*CD133*),^40^ *CD44*,^41^ aldehyde dehydrogenase (ALDH, *ALDH1A1*,^42^ *ALDH1A3*,^43^ *ALDH3A1* ^44^), and *ABCG2* ^45^ (Figure S4B). Among the three ALDHs we examined, 16.8% (162/967) of CSF‐CTCs had detectable expression of *ALDH1A*. 27.7% (268/967) of CSF‐CTCs across five LUAD‐LM patients had *CD44* expression, a stem cell marker for CTC aggregation and polyclonal metastases.^46^ CSF‐CTCs with *PROM1* or *ABCG2* positive were extremely rare. Fifty‐seven CTCs had both *CD44* and *ALDH1A1* expression.

EMT has been suggested as a driver of epithelial tumor spreading.^47^ During the EMT process, epithelial cells lose cell‐cell adhesion and cell polarity in order to gain migration and invasion capabilities to behave like multipotent mesenchymal stem cells.^47^ Almost all CSF‐CTCs had high expression of epithelial markers (Figures 4C and 4D; Figure S4C). However, we discovered a partial EMT process in these CSF‐CTCs, which is defined as tumors cells exhibiting both mesenchymal and epithelial characteristics.^48^ Based on three markers (*FN1*, *VIM*, and *CD44*), 113 CSF‐CTCs in P1 (33.2%) and 54 CSF‐CTCs in P4 (42.5%) had that both epithelial and mesenchymal/CSC markers scores were greater than 0.5 (Figures 4C and 4D), suggesting partial EMT process in these patients. However, other patients only had a few CSF‐CTCs with both epithelial and mesenchymal/CSC characteristics (6 CSF‐CTCs in P2, 1 in P6, and 15 in P7; Figure S4C). Although these CSF‐CTCs had partial EMT features, they lacked expression of N‐cadherin, classical EMT transcription factors (*ZEB1/2, TWIST1/2*, and *SNAIL1/2*), or the EMT regulator TGFβ.^49^

We also examined the ECM‐related markers, which is another class of EMT features. Compared to normal CSF cells, the ECM receptor interaction pathway was significantly enriched in CSF‐CTCs (FDR < 0.05, Figure 2E). We selected core enrichment genes of the ECM receptor interaction pathway, including laminins^50^ (*LAMA3*, *LAMA5*, *LAMB2*, *LAMC1*), integrins^51^ (*ITGA3*, *ITGB4*), and *CD47*.^52^ Abundant expression of ECM genes was observed in all patients, which could be a common feature of CSF‐CTCs (Figures 4C and 4D; Figure S4C). These results suggested that the upregulation of ECM‐related genes might contribute to the generation of CTCs from solid tumor sites or the survival of cancer cells as they circulate in the CSF.

### 3.7 Cancer‐testis antigens in CSF‐CTCs contribute to the among‐patient heterogeneity

Tumor cells frequently express cancer‐testis antigens (CTAs) whose expression is typically restricted to normal male germ cells, providing unprecedented opportunities for clinical development of cancer diagnosis and immunotherapy.^53^ A recent study has demonstrated the extensive heterogeneity of CTAs in LUAD single‐cell data (PDXs and cell lines).^54^ However, little is known about the heterogeneity of all possible CTAs expressed in CSF‐CTCs of LUAD origin. We examined the expression of 276 selected CTAs (http://www.cta.lncc.br/modelo.php) in CSF‐CTCs. We discovered that CTCs from patients P1 and P6 had significantly elevated numbers of expressed CTAs than other patients’ CTCs (Figure 4E), and substantial inter‐tumor heterogeneity and intra‐tumor heterogeneity of CTAs expressed in CSF‐CTCs from five LUAD‐LM patients (Figure 4F). Expression of *XAGE1B* was observed in P1, P6, and P7, whereas *BRDT* expression was restricted in P6 and P7 (Figure 4F). *LY6K* was specific to a subset of CTCs in P4 and P6 (Figure 4F). *SPAG9* was ubiquitously expressed in 41.3% (399/967) of CTCs across five patients at high level (Figure 4F), with the potential to serve as a target for immunotherapy.^55^

### 3.8 Characterization of a case of cancer of unknown primary site through CSF‐CTC single‐cell transcriptomes

**FIGURE 5:**
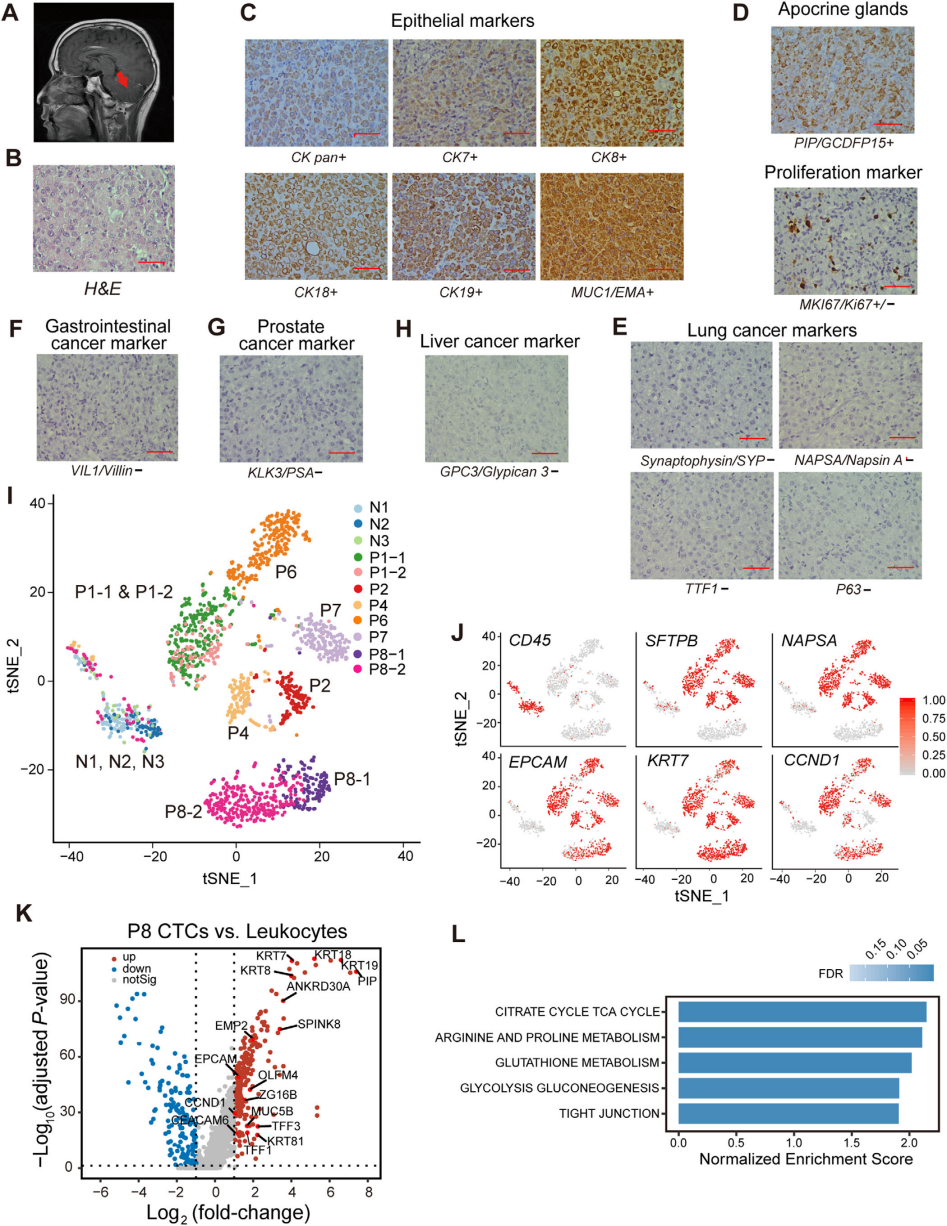
Investigation of a leptomeningeal metastases (LMs) case of cancer of unknown primary site (CUP) through single‐cell RNA sequencing of CSF‐CTCs (cerebrospinal fluid circulating tumor cells). A, Brain MRI (magnetic resonance imaging) result of patient P8, demonstrating leptomeningeal enhancement mainly in the cerebellum (red arrows). B‐H, H‐E (hematoxylin‐eosin) staining (B) and immunohistochemistry (IHC) of biopsy of left cervical lymph nodes for epithelial markers *CK* pan+, *CK7*+, *CK8*+, *CK18*+, *CK19*+, *MUC1*+ (C), apocrine gland marker *PIP*/*GCDFP15*+ and proliferation marker *MKI67*/*KI67*+/– (D), lung cancer markers *NAPSA*–, *TTF*1–, *P63*–, synaptophysin/*SYP*– (E), gastrointestinal cancer marker *VIL1*/Villin– (F), prostate cancer marker *KLK3*/*PAS*– (G), and live cancer marker *GPC3*–/Glypican– (H) (+: positive, –: negative, +/–: partly positive, scale bar represents 200 μm). I, t‐SNE (t‐distributed stochastic neighbor embedding) plot of gene expression clustering of patient P8 with three normal CSF samples (N1, N2, and N3) and five LUAD‐LM (lung adenocarcinoma leptomeningeal metastases) CSF samples (P1, P2, P4, P6, and P7). P8‐1 and P8‐2 are two independent CSF samples 6 months apart. J, Feature plots demonstrating expression of selected genes on the t‐SNE plot (Figure 5A). Scaled expression levels are depicted using a red gradient. *CD45*: leukocyte marker. *SFTPB* and *NAPSA*: lung markers. *EPCAM* and *KRT7*: epithelial marker. *CCND1*: proliferation marker. K, Volcano plot of significant DEGs (differentially expressed genes; adjusted *P*‐value < .05, fold‐change > 2) between P8 CSF‐CTCs and leukocytes. L, Significant enriched KEGG (Kyoto Encyclopedia of Genes and Genomes) pathways by GSEA (gene set enrichment analysis) in P8 CSF‐CTCs compared to leukocytes (FDR < 0.05)

Patient P8, a 49‐year‐old male, was diagnosed with cancer of unknown primary site (CUP) in 2017. CUP is a well‐recognized clinical disorder accounting for 3‐5% of all malignant epithelial tumors; metastatic adenocarcinoma is the most common CUP histopathology (80%).^56^ P8 showed multiple metastases including multiple lymph nodes and leptomeningeal (Figure 5A; Table S1). The hematoxylin‐eosin staining (Figure 5B) and immunohistochemistry (IHC) results (Figure 5C) of biopsy of left cervical lymph nodes indicated metastatic adenocarcinoma positive for epithelial markers (CK pan, *CK7*, *CK8*, *CK18*, *CK19*, and *MUC1*) and a prolactin‐induced protein *PIP/GCDFP15* (Figure 5D, upper panel), which is a small secreted glycoprotein whose expression is generally restricted to cells with apocrine properties.^57^ The proliferation marker *MKI67* was partially positive (Figure 5D, lower panel). Therefore, the primary tumor was epithelial origin with apocrine properties. Based on the IHC results, we could exclude the high possibility of the following locations of the primary tumor: lung cancer (markers *NAPSA−*, *TTF1−*, *P63−*, synaptophysin/*SYP−*; Figure 5E),^58^, ^59^ gastrointestinal cancer (*VIL1*/*Villin−*; Figure 5F),^60^ prostate cancer (*KLK3*/*PAS−*; Figure 5G),^61^ and liver cancer (*GPC−*; Figure 5H).^62^ P8 had partial response to chemotherapy, but the disease recurred with LMs (leptomeningeal enhancement by MRI mainly in cerebellum) in May, 2018 (Figure 5A).

Four hundred ninety‐five CSF cells of P8 remained for analysis based on the same filter criteria as other CSF samples (Table 1). P8 CSF‐CTCs formed a single cluster on the t‐SNE clustering plot, independent from LUAD‐LM CTCs and normal CSF cells (Figure 5I). There was some degree of separation within this cluster between samples P8‐1 and P8‐2, which were collected with a 6‐month time interval, reflecting disease progression (Figure 5I). P8 CSF‐CTCs were defined by the epithelial signature and lack of *CD45* expression (Figure 5J). Consistent with the IHC results (Figure 5E), none of the lung origin markers were expressed (Figures 5J and 5K). In addition, upregulated genes in LUAD‐LM CTCs (for example, *MMP7*, *SCGB3A2*, *C3*, *CDH1*, and *EGFR*) were not detected in P8, except for *CEACAM6*, which was shared across P8 and all LUAD‐LM patients (Figure 5K). The GSEA revealed active metabolism property and tight junction pathway (FDR < 0.05) as the characteristics of P8 CTCs (Figure 5L).

**FIGURE 6:**
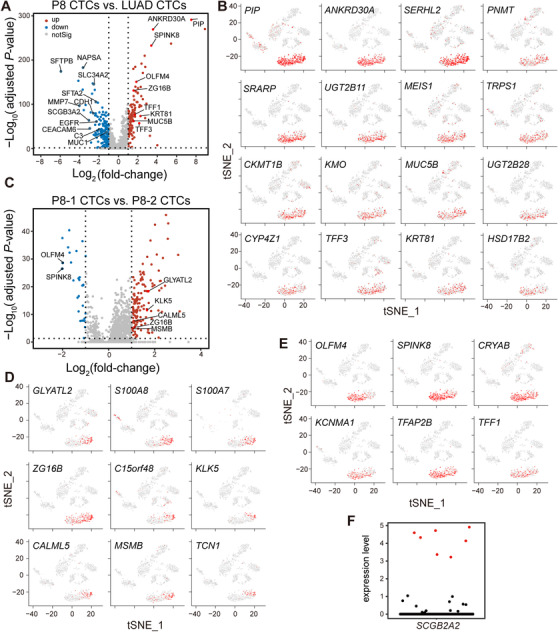
Gene expression profiles and characterization of CSF‐CTCs (cerebrospinal fluid circulating tumor cells) in P8‐1 and P8‐2. A, Volcano plot of differentially expressed genes (DEGs; adjusted *P*‐value < .05) between P8 CSF‐CTCs and LUAD‐LM (lung adenocarcinoma leptomeningeal metastases) CSF‐CTCs. Upregulated (up) and downregulated (down) genes are defined using a fold‐change cutoff of 2. Selected gene names are labeled. B, Feature plots of 16 top P8‐specific genes (selection criteria in Table S5) on the t‐SNE (t‐distributed stochastic neighbor embedding) plot (Figure 5I). Scaled expression levels are depicted using a red gradient (grey denotes lack of expression). C, Volcano plot of DEGs (adjusted *P*‐value < .05) between P8‐1 CSF‐CTCs and P8‐2 CSF‐CTCs. Upregulated (up) and downregulated (down) genes are defined using a fold‐change cutoff of 2. Selected gene names are labeled. D‐E, Feature plots of P8 stage‐biased genes (selection criteria in Table S6) on the t‐SNE plot (Figure 5I). Scaled expression levels are depicted using a red gradient (grey denotes lack of expression). Nine P8‐1 biased genes (D) and six P8‐2 biased genes (E) are plotted. F, Normalized expression levels of *SCGB2A2* in P8 CSF cells (Figure 5I). Seven cells with high expression of *SCGB2A2* are labeled in red

Based on the single‐cell transcriptome profiles, 40 P8 cluster defining genes were selected according to the selection criteria listed in Table S5 and Table S6. Among those genes, the top two candidates were *PIP* and *ANKRD30A* (Figures 6A and 6B). *PIP* is a cytoplasmic marker commonly used to identify breast cancer, but not exclusively, as its expression is also found in several other types of human cancers including prostate, sweat, and salivary gland cancer.^57^ *ANKRD30A* is restricted to normal breast, normal testis, normal prostate and also detected in breast cancer as a breast cancer‐specific marker and in prostate cancer.^63^, ^64^ It is very interesting that seven P8 CTCs had high expression of the *SCGB2A2* gene (Figure 6F), a carcinoma marker of breast origin including primary tissues, metastatic tissues, and blood‐CTCs.^65^, ^66^ *SCGB2A2* is also positive in some tissues of gynecologic malignancies,^67^ but P8 is a male. In addition, *SCGB2A2* is also associated with salivary gland cancer.^68^

Two hundred two genes were differentially expressed between P8‐1 and P8‐2 CTCs (Figure 6C). Nine genes were preferentially expressed in P8‐1 CTCs (Figure 6D; Table S6), whose expression could not be detected in most CTCs at the later stage P8‐2. *OLFM4, SPINK8, CRYAB, KCNMA1*, *TFAP2B*, and *TFF1* were significantly upregulated during tumor progression in P8‐2 CTCs (Figure 6E; Table S6).

The proliferation ability of P8 CTCs was similar to LUAD‐LM CSF‐CTCs, with three of 131 (P8‐1) and eleven of 262 (P8‐2) cells in high‐cycling state (Figure S5A). Interestingly, a large proportion of (118/393) P8 cells had *PROM1* (*CD133*, a classic CSC marker) expression, which was different from LUAD CSF‐CTCs (20/967). All P8 CTCs had high expression of epithelial genes (Figure S5B). 73.8% (290/393) of P8 CTCs were in stem‐like phenotype (*PROM1+CD44*), and 9.2% (36/393) of P8 CTCs had detectable expression of mesenchymal genes (*FN1+VIM*) (Figure S5B).

## 4 DISCUSSION

CTCs and cell‐free DNA (cfDNA) in CSF samples can reflect the real‐time status of leptomeningeal disease and have promising potential for characterization and monitoring of LM development. However, false negatives in CTC capturing^11^ and false positives of cfDNA mutations,^69^ have limited the application of CSF on the diagnosis and therapy of LMs. In this study, we characterized the single‐cell transcriptome profiles of CSF‐CTCs by scRNA‐seq for the first time to facilitate the early detection of LMs and the identification of potential therapeutic targets.

### 4.1 The transcriptome characteristics of CSF‐CTCs from LUAD‐LM patients

#### 4.1.1 Heterogeneity of CSF‐CTCs

One major advantage of single‐cell RNA‐seq approach is the ability to characterize the expression variation among individual cells. Our study found significant among‐patient heterogeneity and among‐cell heterogeneity within a given patient, which could be explained by spatial heterogeneity of metastatic sites, cell‐cycle gene, and CTA expression profiles, as well as the proportion of CTCs displaying mesenchymal and CSC properties. In addition, the temporal difference during disease progression and patient‐specific mutations also contributed to the heterogeneity among CTCs, suggesting the need of personalized diagnosis and expression profiling.

##### Temporal heterogeneity

The scRNA‐seq approach could detect potential temporal heterogeneity during tumor progression. We obtained CSF samples from two different time points for both patient P1 (P1‐1 vs P1‐2) and P8 (P8‐1 vs P8‐2). Collected within a 2‐month time interval, P1‐1 and P1‐2 CSF‐CTCs from two datasets formed a single homogeneous cluster (Figure 2A), indicating similar transcriptome patterns. In contrast, P8‐1 and P8‐2 were collected 6‐month apart as the patient's condition worsened significantly. Although they were still in the same cluster, we observed some degree of separation on the t‐SNE plot (Figure 5I). The time interval of sample collection is one reason for the clustering difference between two samples of P1 and P8. P1 LMs originated from LUAD, and instead P8 was CUP‐LM. The disease progress of LUAD‐LM or CUP‐LM is different, also contributing to the clustering difference. In addition, P1 is still alive until now with better prognosis than P8, who showed obvious disease progress from the beginning of 2019 and died in August, 2019. Systematic sampling over a time course in future studies will allow better characterization of the temporal heterogeneity.

##### Mutational profile heterogeneity and other types of heterogeneity

The mutational profiles of tumor cells might also contribute to the observed heterogeneity. Mutations in CSF cfDNA (CSF cfDNA) have been detected by next‐generation sequencing (Table S4). The discovery of common driver mutations and the development of targeted therapies have dramatically improved the treatment efficacy of intracranial tumors and prolonged survival. Activating mutations of the *EGFR* (epidermal growth factor receptor) gene and *ALK* (anaplastic lymphoma kinase) rearrangements are keys in the development of BMs.^70^, ^71^ *EGFR* (19del) and *ALK* (Arg1192Trp) mutations, along with *TP53* (Trp53Ter) mutation and low frequency of *KRAS* mutation were detected in P1 CSF cfDNA (Table S4). *EGFR* (Leu858Arg) mutation and *TP53* (Arg248Gln) mutation were found in P4 CSF cfDNA, and P7 CSF cfDNA only exhibited *TP53* (Asp281Tyr) mutation (Table S4). We have not performed CSF cfDNA mutation detection by NGS in P2 or P6, but the *EGFR* mutation in P6 tumor was detected by other method in 2012. These mutational profiles guided different targeted therapy strategies, especially for *EGFR*. In summary, the patient clinical characteristics (Table S1) including age, sex, duration of disease, primary LUAD site, metastatic sites in LMs (Figure 3A) or other tissues and organs, therapies received before sample collection and tumor mutational profile (Table S4), were different and comparable, contributing to observed transcriptome heterogeneity of CSF‐CTCs among five LUAD‐LM patients.

#### 4.1.2 The metastatic potential of CSF‐CTCs

EMT is a process related to tumor invasion and metastases. It has been reported that some NSCLC blood‐CTCs have a dual epithelial‐mesenchymal phenotype.^72^ Similarly, we discovered abundant expression of epithelial genes in LUAD‐LM CSF‐CTCs, and a small subset of CSF‐CTCs expressed mesenchymal genes (Figures 4C and 4D; Figure S4B‐C). However, LUAD‐LM CSF‐CTCs with high expression of mesenchymal genes and low expression of epithelial genes were extremely rare (only two cells shown in Figure S4D), which is a major difference compared to NSCLC blood‐CTCs. As an important stem cell marker of CSF‐CTCs, CD44 improves tumor initiation capacities of CTCs.^46^ We did not observe any correlations between CD44 expression level and enrichment for the mesenchymal genes (*VIM* and *FN1*) within single CSF‐CTC (Figure S4E), suggesting that stem cell markers and EMT markers were not intrinsically linked in CSF‐CTCs. Similar results have also been observed in pancreatic blood‐CTCs.^73^ The advancement of CSF‐CTC metastatic characteristics and the comparison with NSCLC blood‐CTCs will provide a much better understanding of the mechanisms of LUAD‐LM.

Notably, we also identified the unexpected abundant expression of ECM genes in CSF‐CTCs (Figures 4C and 4D), consistent with ECM characteristics of blood‐CTCs in pancreatic, breast, and prostate origin.^73^ Tumor stroma‐derived ECM signaling plays an important role in targeting cancer cell metastasis.^74^ The cell‐autonomous expression of ECM genes in CSF‐CTCs may contribute to the dissemination of cancer.

#### 4.1.3 Adhesion pathways were significantly enriched in LUAD CSF‐CTCs

Endothelial cells are the main component of the blood‐brain barrier (BBB), and the disruption of tight junctions between endothelial cells by disease or drugs can compromise the leptomeningeal.^75^ *MMP7* (log_2_FC = 3.99; Figure 2D) has been reported to open the BBB by degrading tight junctions proteins.^76^ *CLDN7* (claudin‐7; Table S7; log_2_FC = 2.89) is another adhesion‐related gene, which was greatly upregulated in CSF‐CTCs. Claudins are solely involved in tight junctions and critical for cell‐to‐cell adhesion in epithelial cells, *CLDN2* and *CLDN7* have been shown to facilitate the adhesion of cancer cells to the ECM, which is important for cancer metastasis.^77^, ^78^ The roles of *CLDN7* in LUAD‐LM are still unknown. Whether *CLDN7*‐mediated adhesion to the BBB is advantageous for LUAD‐LM warrants further study.

Intercellular adhesion molecule‐1 (*ICAM1*; Table S7; log_2_FC = 2.12) was also significantly upregulated in most LUAD‐LM CSF‐CTCs. *ICAM1*, a member of an immunoglobulin‐like superfamily of adhesion molecules, is involved in various processes in lung cancer development and signal transduction across leukocyte‐epithelial cell interactions.^79^ It has been reported that the formation of CTC‐WBC clusters (mainly neutrophils) benefits the circulation and invasion of breast‐CTCs. A related adhesion molecule, *VCAM1*, mediates the interaction between breast‐CTCs and neutrophils.^80^ Future research is needed to reveal whether *ICAM1* contributes to the formation of CSF‐CTC‐WBC clusters in LUAD‐LM patients. *MMP7*, *CLDN7*, and *ICAM1* have great potential as therapy targets to decrease the metastatic ability of CSF‐CTCs.

### 4.2 Candidate genes for an RNA‐based digital detection of CSF‐CTCs in LUAD‐LM

Seventy‐eight genes highly expressed in CSF‐CTCs were selected for diagnostic purposes based on the selection criteria shown in Table S7. These genes include epithelial markers (*CDH1, EPCAM, KRT18, KRT7, KRT8, MUC1*), lung origin markers (*SFTPB, NAPSA, SFTA2*,^81^ *SLC34A2*,^82^ and *EMP2* ^83^), secreted proteins (*CEACAM6* and *SCGB3A2*), blood‐CSF barrier‐associated genes (*MMP7* and *C3*), and cell‐cycle genes *CCND1* (Figures 2B and 2D; Table S7). In addition, epidermal growth factor receptor^84^ (*EGFR* and *ERBB2*) and adhesion‐related genes (*CLDN7* and *ICAM1*) also correspond to the characteristics of LUAD‐LM CSF‐CTCs (Figure 2D; Table S7). An initial set of 20 genes is chosen as a panel for an RNA‐based molecular signature of CSF‐CTCs, which has great potential in clinical LUAD‐LM diagnosis with a sufficient sensitivity and specificity.

### 4.3 The power of scRNA‐seq in CSF samples for the diagnosis of CUP origin

CUP patients commonly have poor prognosis due to treatment with a non‐selective empirical therapy.^56^ Identification of the primary tumor type will greatly inform treatment strategies, but it is extremely challenging. Our studies enrolled one CUP patient of metastatic adenocarcinoma (P8).

In order to pinpoint the origin of P8's malignancy, patient history, physical examination, serum markers, histological data, and state‐of‐the‐art imaging results were examined, but the primary origin remained inconclusive. For CUP patients with LM only, the CSF‐CTCs are the available tissue samples for the diagnosis of the primary origin of CUP. Since P8 was a CUP case with multi‐site metastases, we had the biopsy of left lymph nodes to perform IHC to pair with our scRNA‐seq data. The scRNA‐seq data of CSF‐CTCs and IHC results of biopsy revealed an epithelial origin and low possibilities to be of lung, prostate, gastrointestinal, and liver origin, providing crucial diagnostic information for patient P8. The cluster‐defined genes, *PIP* and *ANKRD30A* (Figures 6A and 6B), were exclusively expressed in P8 CSF‐CTCs, indicating sufficient evidence to diagnose the primary site as breast cancer, sweat/salivary gland cancer, or prostate cancer. Interestingly, when we evaluated the expression of *SCGB2A2* (a classical marker of breast cancer), seven CTCs from P8 had high expression levels (Figure 6F), whereas other CSF‐CTCs had little to no expression, showing the advantage of scRNA‐seq over bulk RNA‐seq or IHC. This scRNA‐seq result enhanced the diagnosis directions of breast cancer or sweat/salivary gland cancer origin. Further investigations were made on the possibility of breast or sweat/salivary gland cancer, but no evidences were found despite of extensive imaging examinations. A definitive conclusion could not be made because P8 had passed away and refused autopsy.

As the first CUP case with scRNA‐seq data of CSF‐CTCs, we were able to achieve a comprehensive characterization of the transcriptome pattern in every P8 tumor cell, as well as the discovery of potential biomarkers expressed at a low frequency in specific cells. With continuous advancement of scRNA‐seq technology and decrease of sequencing cost, additional scRNA‐seq datasets will be available for breast cancer and sweat/salivary gland cancer, providing the possibility to define the origin of P8 LMs. In the near future, based on the development of single‐cell transcriptome databases of multiple CUP cases, we will be able to provide speedy and accurate diagnosis for CUP origin to benefit this category of cancer patients. The discussion of CSF‐CTCs transcriptome signature in P8 CUP case is shown in Supplementary Discussion.

## 5 CONCLUSION

In summary, we defined CSF‐CTCs from five LUAD‐LM patients and one CUP‐LM patient, and showed their single‐cell transcriptome characteristics of marker genes, abilities of proliferation and metastasis, and great heterogeneity, which have provided a new direction for the diagnosis and therapy of LMs. Our study is the first one to focus on CSF‐CTCs. In the future, we intend to establish an RNA‐based digital detection of CSF‐CTCs to help diagnose LUAD‐LMs and focus on the interactions between the CSF microenvironment and CSF‐CTCs.

## CONFLICT OF INTEREST

The authors declare that there is no conflict of interest that could be perceived as prejudicing the impartiality of the research reported.

## AUTHOR CONTRIBUTIONS

Haoyu Ruan designed the project. Haoyu Ruan, Ying Xu, Linyu Pi, and Weizhe Ma conducted the experiments and sequencing. Haoyu Ruan, Yihang Zhou, Yue Zhai, and Jie Shen performed the bioinformatic analyses. Haoyu Ruan, Kun Chen, and Xiangyu Li collected clinical samples. Zhiyuan Wu and Xuan Deng contributed to the design of the project. Haoyu Ruan and Yihang Zhou wrote the manuscript with help from all authors. Xu Wang, Chao Zhang, and Ming Guan supervised and coordinated all aspects of the research.

## DATA AND MATERIALS AVAILABILITY

The data generated in this study have been submitted to the NCBI BioProject database (https://www.ncbi.nlm.nih.gov/bioproject/) under accession number PRJNA602172. All data generated or analyzed during this study are included in this published article. All the data are available from the corresponding author upon reasonable request.

## Supporting information

SUPPORTING INFORMATION
