# Supplementary material for: Circulating tumor cell characterization of lung cancer brain metastases in the cerebrospinal fluid through single‐cell transcriptome analysis
Source: Clin Transl Med. 2020 Dec 15;10(8):e246. doi: 10.1002/ctm2.246 (PMC7737787; doi:10.1002/ctm2.246)
Supplement: Supplementary file 1 — SUPPORTING INFORMATION [file CTM2-10-e246-s001.pdf]

**FIGURE S1** Fluorescence-activated cell sorting (FACS) of CSF-CTCs (cerebrospinal fluid circulating tumor cells) from LUAD-LM (lung adenocarcinoma leptomeningeal metastases) patient samples. CTCs lacking CD45 (APC-A) expression and showing larger FSC-H than normal leukocytes. Calcein Blue AM (UltraViolet-A excitation) was used to select live cells.

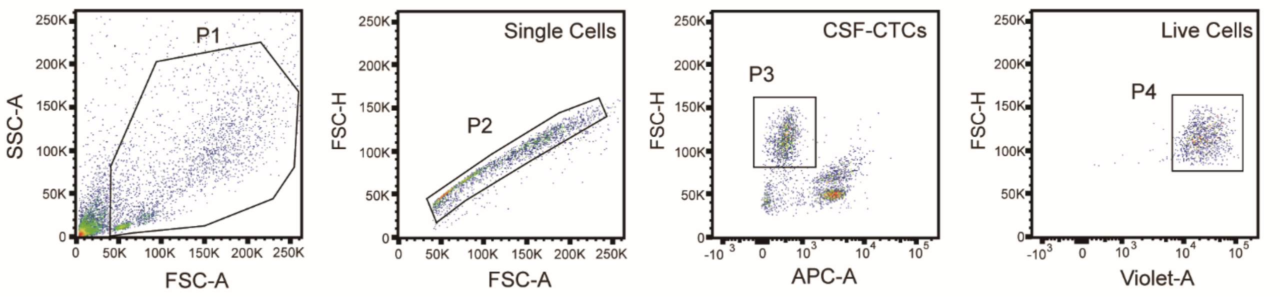

**FIGURE S2** t-SNE plots of LUAD-LM (lung adenocarcinoma leptomeningeal metastases) patients' CSF-CTCs (cerebrospinal fluid-circulating tumor cells) based on single-cell transcriptome profiles. **A**, t-SNE (t-distributed stochastic neighbor embedding) plot of CSF-CTCs clustering of five LUAD-LM CSF samples with a minimum number of covered genes of 1,000. **B**, Three groups of different covered genes number (1000-1999, 2000-2999, and 3000-3999) are highlighted for each cell on the t-SNE plot (***Figure S2A***). **C**, t-SNE clustering plot of LUAD-LM patient P3 with three normal CSF samples (N1, N2 and N3) and five LUAD-LM patient CSF samples (P1, P2, P4, P6 and P7). P3 CSF sample was processed following the protocol in ***Figure 1A***. Candidate CTCs were selected merely based on cell morphology without CD45 negative cell selection. Cell identity analysis revealing that all cells collected from P3 CSF sample are monocytes.

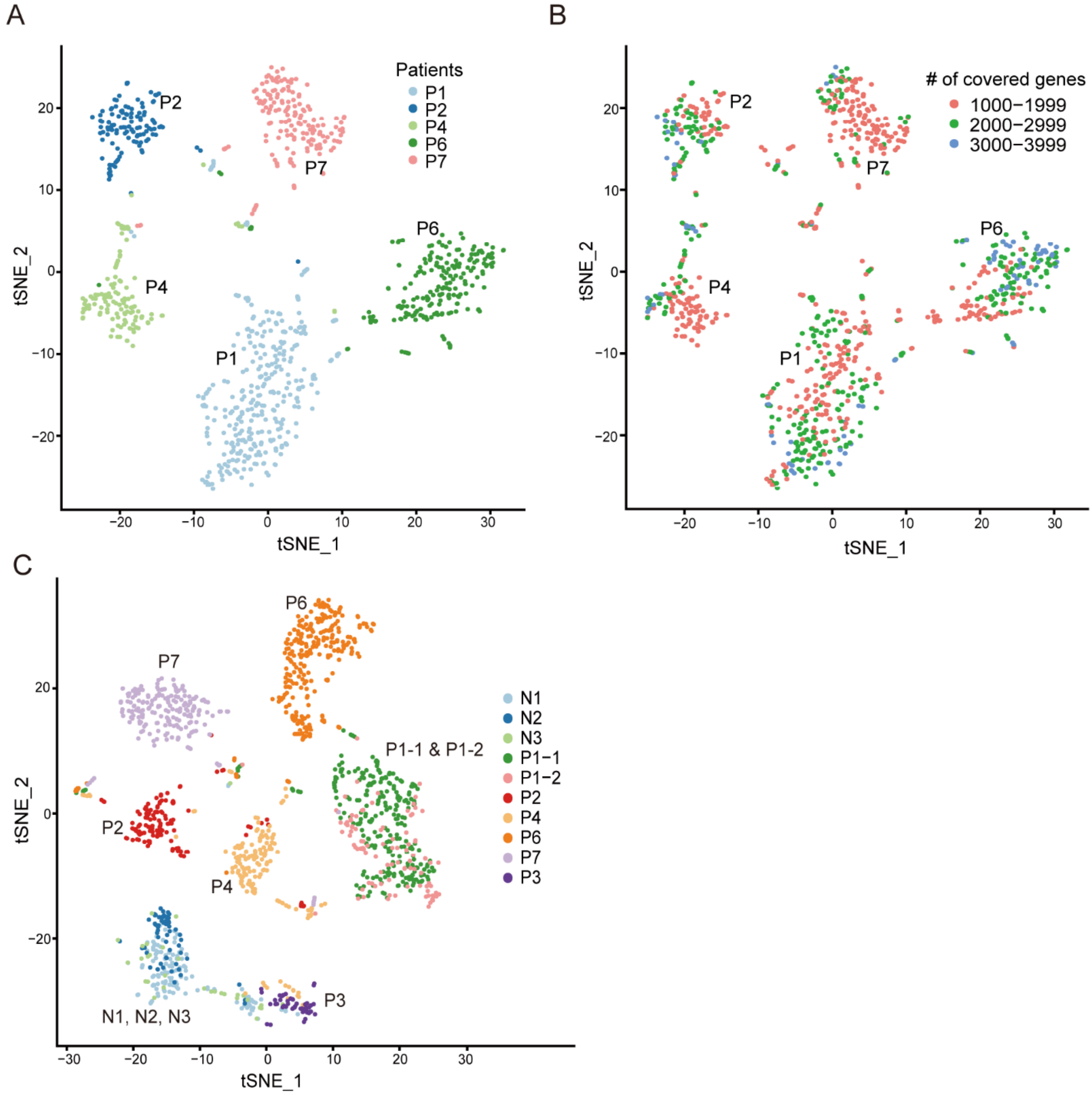

**FIGURE S3** Feature plots demonstrating the expression of the following selected genes on the t-SNE (t-distributed stochastic neighbor embedding) plot (***Figure 2A***). Scaled expression levels are depicted using a red gradient (grey denotes lack of expression). Immune marker genes: *PTPRC*, *CD2*, *CD3D*, *CD3E*, *CD3G*, *CD14*, *CD68* and *CD163*. Lung associated genes: *SFTPA1*, *SFTPA2*, *SFTPB* and *NAPSA*. Epithelial marker genes: *EPCAM*, *CDH1*, *KRT7*, *KRT8*, *KRT17*, *KRT18*, *KRT19* and *MUC1*. Cell cycle associated genes: *CCND1* and *TOP2A*.

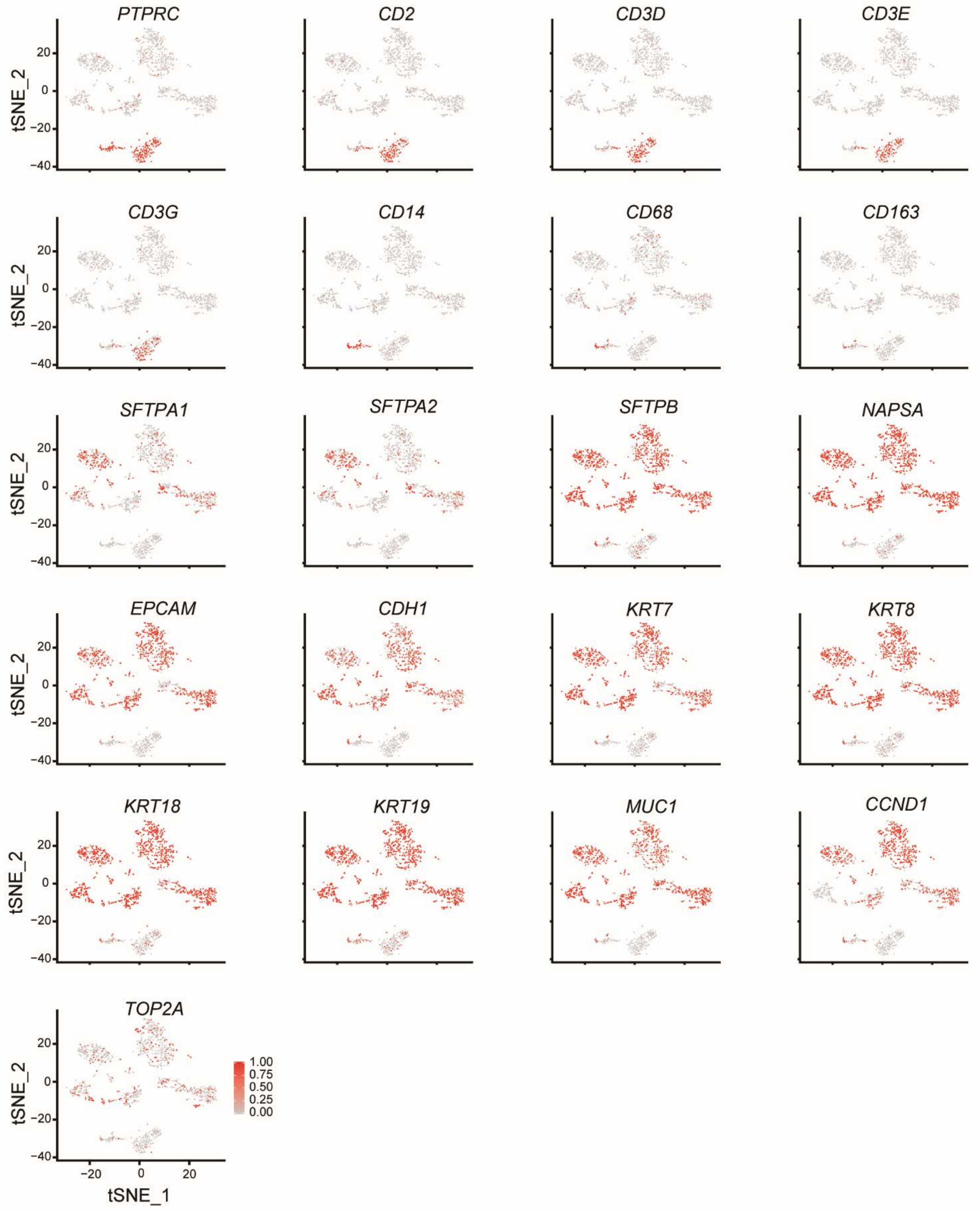

**FIGURE S4** Gene expression profiles of cell cycle genes, CSC (cancer stem cell) genes and genes related to partial EMT (epithelial-to-mesenchymal transition) in CSF-CTCs (cerebrospinal fluid circulating tumor cells). **A**, Estimation of the cell cycle state of individual CTCs (dots) in P2, P6, P7, H358 cell line and PT45 based on the relative expression of G1/S (x axis) and G2/M (y axis) gene sets. Cells are colored as that in ***Figure 4A***. **B**, Feature plots demonstrating the expression of CSC markers (*PROM1*, *CD44*, *ALDH1A1*, *ALDH1A3* and *ALDH3A1*) on the t-SNE (t-distributed stochastic neighbor embedding) plot (***Figure S2A***). Scaled expression levels are depicted using a red gradient (grey stands for lack of expression). **C**, Unsupervised clustering based on GSVA (gene set variation analysis) score (*top*) and average normalized expression level (*bottom*) of epithelial (Epi) gene set, Mes/CSC (mesenchymal/cancer stem cell) gene set and extracellular matrix (ECM) gene set in CSF-CTCs from patient P2, P6 and P7. **D**, Scatterplot of average expression value of epithelial (Epi) genes on the *x*-axis and mesenchymal (Mes) genes on the *y*-axis for LUAD-LM (lung adenocarcinoma-leptomeningeal metastases) CSF-CTCs (dots). The two cells with high expression of mesenchymal (Mes) genes and low expression of epithelial (Epi) genes are labeled in red. **E**, Scatterplot of average expression value of mesenchymal (Mes) genes on the *x*-axis and *CD44* on the *y*-axis for LUAD-LM CSF-CTCs (dots). The gene sets used in ***Figure S4C-E*** are same as those in ***Figure 4C***.

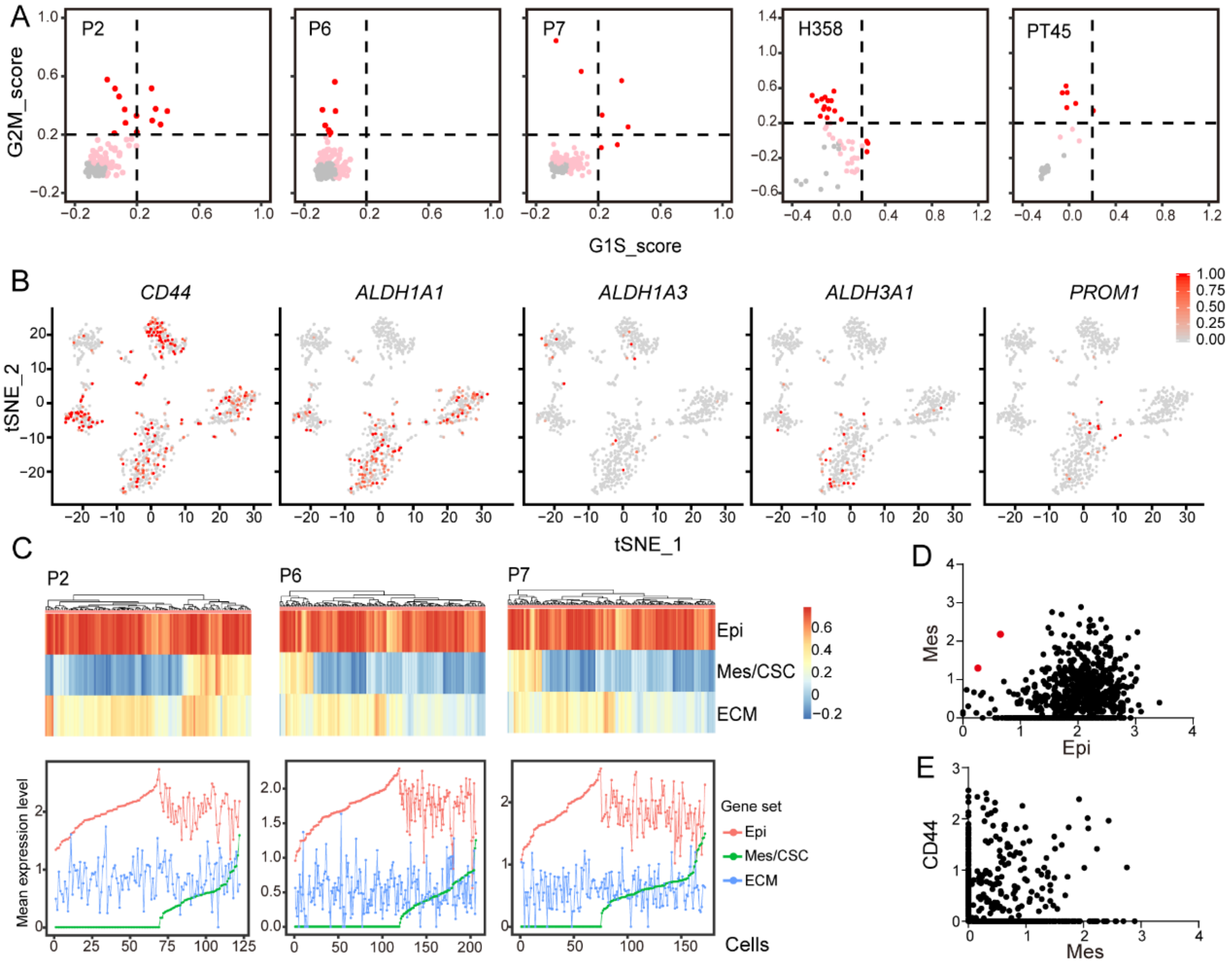

**FIGURE S5** Gene expression profiles of cell cycle genes, epithelial and mesenchymal genes and CSC (cancer stem cell) genes in P8 CSF-CTCs (cerebrospinal fluid circulating tumor cells). **A**, Cell cycle state of individual CSF-CTCs (dots) inferred from relative expression of G1/S (*x*- axis) and G2/M (*y*-axis) gene sets in P8-1 and P8-2 samples. Cells are colored by inferred cell cycle states (cycling cells: score > 0.2, red; intermediate cells: 0 < score ≤ 0.2, pink; noncycling cells: score ≤ 0, gray). **B**, Average normalized expression level (*y*-axis) of epithelial genes (*EPCAM*, *KRT18*, *KRT19*, *KRT7*, *KRT8*, *MUC1*), mesenchymal genes (*VIM*, *FN1*) and CSC genes (*PROM1*, *CD44*) in P8 CSF-CTCs (*x*-axis).

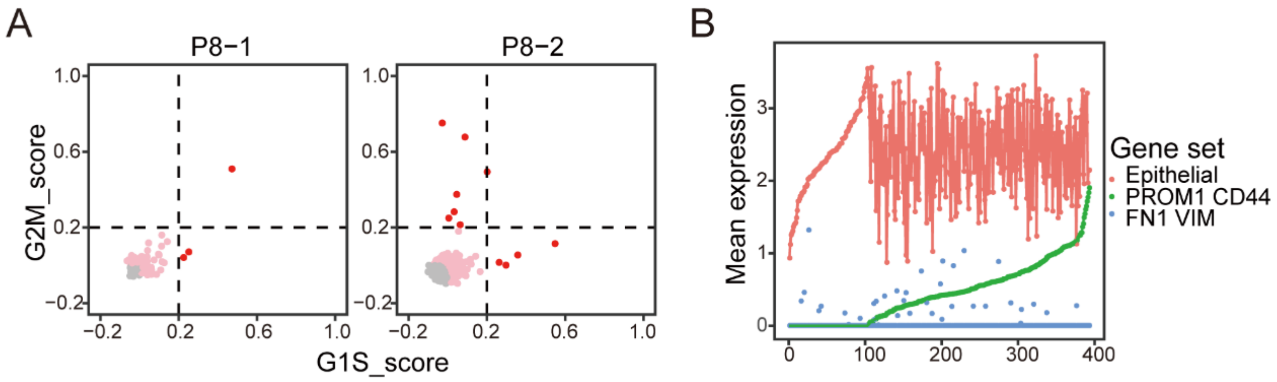

**TABLE S1** Clinical information and cell selection conditions for CSF and blood samples.

| Patient ID/info | Age | Gender | LUAD diagnosis time | LM diagnosis time | Primary LUAD site | Metastatic sites in leptomeningeal by MRI | Metastatic sites in other tissues and organs | Therapies received before sample collection | Sample collection date | Markers for FACS sorting | Number of cells sequenced |
|---|---|---|---|---|---|---|---|---|---|---|---|
| **P1 LUAD** | 36 | Male | 11/2017 | 01/2018 | Left lung | Left frontal lobe, Right parietal lobe | Right lung, pleura, multiple lymph nodes, intercostal muscles, bone, brain, | Chemotherapy and targeted therapy | 11/1/2018 (P1-1)<br>1/7/2019 (P1-2) | Calcein Blue AM+ CD45– | 168<br>360 |
| **P2 LUAD** | 62 | Female | 03/2018 | 12/2018 | Left lung | Bilateral temporal lobe | N/A | N/A | 12/17/2018 | Calcein Blue AM+ CD45– | 192 |
| **P4 LUAD** | 62 | Female | 12/2017 | 02/2019 | Right lung | Cerebellum | Bone | Chemotherapy and targeted therapy | 3/1/2019 | Calcein Blue AM+ CD45– | 288 |
| **P6 LUAD** | 55 | Male | 12/2012 | 03/2019 | Right lung | Bilateral temporal lobe | N/A | Chemotherapy and targeted therapy | 3/8/2019 | Calcein Blue AM+ CD45– | 480 |
| **P7 LUAD** | 60 | Male | 07/2017 | 05/2019 | Right lung | / | N/A | Chemotherapy | 5/10/2019 | Calcein Blue AM+ CD45– | 288 |
| **P3 LUAD** | 49 | Male | 02/2017 | 02/2018 | Right lung | Right temporal lobe | N/A | Chemotherapy and targeted therapy | 3/3/2019 | Calcein Blue AM+ | 96 |
| **P8 CUP** | 49 | Male | 12/2017 | 05/2018 | N/A | Cerebellum and Cerebral hemisphere | multiple lymph nodes | Chemotherapy | 1/14/2019 (P8-1)<br>6/21/2019 (P8-1) | Calcein Blue AM+ CD45 | 480<br>816 |
| **N1 control** | 40 | Male | N/A | N/A | N/A | N/A | N/A | N/A | 10/8/2018 | Calcein Blue AM+ | 288 |
| **N2 control** | 35 | Female | N/A | N/A | N/A | N/A | N/A | N/A | 10/10/2018 | Calcein Blue AM+ | 240 |
| **N3 control** | 39 | Male | N/A | N/A | N/A | N/A | N/A | N/A | 10/18/2018 | Calcein Blue AM+ | 96 |
| **Blood T cells** | 27 | Female | N/A | N/A | N/A | N/A | N/A | N/A | 1/25/2019 | Calcein Blue AM+ CD45+ CD3+ | 168 |
| **Blood B cells** | 27 | Female | N/A | N/A | N/A | N/A | N/A | N/A | 1/25/2019 | Calcein Blue AM+ CD45+ CD19+ | 168 |

CSF: cerebrospinal fluid; LUAD: lung adenocarcinoma; CUP: cancer of unknown primary site; LM: leptomeningeal metastases; FACS: fluorescence activating cell sorter; Calcein Blue AM: labeling dye for live cells selection; CD45: protein tyrosine phosphatase receptor type C, marker for leukocytes; CD19: marker for B cells; CD3: marker for T cells; N/A: Not applicable; “ / ”: no leptomeningeal enhancement shown by MRI (magnetic resonance imaging).

**TABLE S2** Summary of cell type identity of cells in normal CSF samples and blood samples.

| Sample ID | Total filtered cells | T cells | B cells | Monocytes |
|---|---|---|---|---|
| N1 | 127 | 105 | | 22 |
| N2 | 53 | 51 | | 2 |
| N3 | 27 | 14 | | 13 |
| Blood T | 41 | 41 | | |
| Blood B | 41 | | 41 | |
| **Total** | **289** | **211** | **41** | **37** |

**TABLE S3** Summary of sequenced reads and detected genes of cells in CSF (cerebrospinal fluid) samples and blood samples.

| Sample ID | # of sequencing reads | # of filtered reads* | # of uniquely mapped reads | Average # of uniquely mapped reads per cell | Total genes detected | # of genes whose counts>1000 |
|---|---|---|---|---|---|---|
| **P1-1** | 58380064 | 15206906 | 6734715 | 40088 | 11655 | 436 |
| **P1-2** | 237321692 | 42055297 | 16346993 | 45408 | 13813 | 1412 |
| **P2** | 147219734 | 33865056 | 12336410 | 64252 | 12893 | 652 |
| **P4** | 350537537 | 15235749 | 6579460 | 22845 | 13518 | 1203 |
| **P6** | 302888314 | 123455881 | 47199598 | 98332 | 14262 | 1692 |
| **P7** | 94506564 | 24521678 | 7850335 | 27258 | 12170 | 371 |
| **P3** | 89980565 | 6222803 | 2340870 | 24384 | 9243 | 233 |
| **P8-1** | 297958378 | 74043518 | 35977243 | 74953 | 13340 | 2012 |
| **P8-2** | 329258208 | 73341499 | 27047520 | 70436 | 13340 | 1332 |
| **N1** | 125071290 | 27453376 | 6834185 | 23730 | 13592 | 308 |
| **N2** | 224306614 | 49201160 | 5036090 | 26230 | 10944 | 236 |
| **N3** | 105595344 | 12538866 | 3107257 | 32367 | 10101 | 90 |
| **Blood T** | 91515818 | 22523435 | 9798201 | 58323 | 7522 | 428 |
| **Blood B** | 104966890 | 25259710 | 11184723 | 66576 | 9084 | 484 |

*: *filtered reads*: demultiplex combined sequenced reads via barcodes; removed poly-T bases in forward reads using manual scripts; for each read, extracted barcode and expand read name with it; remove low-quality bases and reads using *Trim galore!* setting -q=25 --phred33 --stringency=3. *mapping parameters*: reference genome is hg38; default parameters of STAR alignment.

**TABLE S4** Summary of disease relevant mutations detected in cerebrospinal fluid cell free DNA (CSF-cfDNA) by next-generation sequencing.

| Patient ID | Sample collection date | EGFR | TP53 | ALK | KRAS(P21) |
|---|---|---|---|---|---|
| P1 | 20180122 | p.Glu746_Ala750del (25.34%) | p.Trp53Ter (85.47%) | p.Arg1192Trp (82.66%) | p.Gln61Leu (0.13%), p.Gly138Arg (0.20%) |
| P2 | N/A | | | | |
| P4 | 20190307 | p.Leu858Arg (16.09%) | p.Arg248Gln (44.72%) | | |
| P6 | N/A | | | | |
| P7 | 20190510 | | p.Asp281Tyr (89.72%) | | |

p: protein; numbers in brackets indicate mutation frequencies; N/A, Not applicable.

**TABLE S5** List of top 25 genes defining patient P8 cluster.

| gene | Average $log_2FC$ | adjusted *P*-value | pct in P8 | pct in others | pct diff |
|---|---|---|---|---|---|
| *PIP* | 7.496 | 0.000 | 0.945 | 0.050 | 0.895 |
| *ANKRD30A* | 3.423 | 0.000 | 0.794 | 0.007 | 0.787 |
| *SERHL2* | 2.550 | 0.000 | 0.699 | 0.034 | 0.665 |
| *PNMT* | 2.620 | 0.000 | 0.606 | 0.010 | 0.596 |
| *SRARP* | 1.415 | 0.000 | 0.554 | 0.000 | 0.554 |
| *UGT2B11* | 2.201 | 0.000 | 0.400 | 0.004 | 0.396 |
| *MEIS1* | 1.281 | 0.000 | 0.400 | 0.025 | 0.375 |
| *TRPS1* | 0.779 | 0.000 | 0.386 | 0.025 | 0.361 |
| *CKMT1B* | 0.749 | 0.000 | 0.400 | 0.042 | 0.358 |
| *KMO* | 0.688 | 0.000 | 0.335 | 0.015 | 0.320 |
| *MUC5B* | 1.478 | 0.000 | 0.313 | 0.026 | 0.287 |
| *UGT2B28* | 0.784 | 0.000 | 0.279 | 0.000 | 0.279 |
| *CYP4Z1* | 0.705 | 0.000 | 0.281 | 0.002 | 0.279 |
| *TFF3* | 2.027 | 0.000 | 0.309 | 0.031 | 0.278 |
| *KRT81* | 2.123 | 0.000 | 0.287 | 0.009 | 0.278 |
| *HSD17B2* | 0.587 | 0.000 | 0.212 | 0.006 | 0.206 |
| *HPX* | 0.603 | 0.000 | 0.216 | 0.021 | 0.195 |
| *CCDC160* | 0.635 | 0.000 | 0.196 | 0.003 | 0.193 |
| *MOV10L1* | 0.585 | 0.000 | 0.194 | 0.020 | 0.174 |
| *MUC5AC* | 0.928 | 0.000 | 0.174 | 0.003 | 0.171 |
| *ZCCHC2* | 0.906 | 0.000 | 0.172 | 0.031 | 0.141 |
| *EFHD1* | 0.700 | 0.000 | 0.139 | 0.025 | 0.114 |
| *DCD* | 3.152 | 0.000 | 0.111 | 0.000 | 0.111 |
| *IGKC* | 2.774 | 0.000 | 0.121 | 0.041 | 0.080 |
| *R3HCC1L* | 0.651 | 0.019 | 0.111 | 0.049 | 0.062 |

Patient P8 cluster defining genes were selected compared to other CSF (cerebrospinal fluid) samples (***Figure 5I***) by fold-change FC > 1.5, pct in others ≤ 0.05 and adjusted *P*-value < 0.05; pct in P8: the percentage of cells where the gene is detected in P8 CSF samples; pct in others: the percentage of cells where the gene is detected in other CSF samples; pct diff: the difference between the percentage of cells where the gene is detected in P8 CSF sample and that in other CSF samples (P8 minus others).

P8 cluster defining genes with pct dff > 0.2 are shown in ***Figure 6B***.

**TABLE S6** List of patient P8 cluster defining genes with significant biased expression in P8-1 or P8-2 circulating tumor cells (CTCs).

| **gene** | **avg $log_2$FC** | **adjusted *P*-value** | **pct in P8** | **pct in others** | **pct in P8-1** | **pct in P8-2** | **adjusted *P*-value** | **avg $log_2$ FC** | **pct diff** |
|---|---|---|---|---|---|---|---|---|---|
| *GLYATL2* | 1.219 | 0.000 | 0.196 | 0.011 | 0.486 | 0.082 | 0.000 | 1.708 | 0.404 |
| *KLK5* | 0.853 | 0.000 | 0.109 | 0.001 | 0.297 | 0.031 | 0.000 | 1.657 | 0.266 |
| *ZG16B* | 1.435 | 0.000 | 0.426 | 0.003 | 0.662 | 0.386 | 0.000 | 1.093 | 0.276 |
| *S100A8* | 2.488 | 0.000 | 0.293 | 0.017 | 0.568 | 0.171 | 0.000 | 0.639 | 0.397 |
| *S100A7* | 2.382 | 0.000 | 0.214 | 0.004 | 0.493 | 0.109 | 0.000 | 0.830 | 0.384 |
| *MSMB* | 0.855 | 0.000 | 0.188 | 0.005 | 0.372 | 0.126 | 0.000 | 1.021 | 0.246 |
| *C15orf48* | 0.603 | 0.000 | 0.188 | 0.023 | 0.385 | 0.113 | 0.000 | 0.915 | 0.272 |
| *CALML5* | 0.792 | 0.000 | 0.158 | 0.004 | 0.351 | 0.085 | 0.000 | 1.090 | 0.266 |
| *TCN1* | 0.787 | 0.000 | 0.115 | 0.003 | 0.264 | 0.055 | 0.000 | 1.102 | 0.209 |
| *SPINK8* | 3.245 | 0.000 | 0.703 | 0.002 | 0.486 | 0.918 | 0.000 | -2.022 | -0.432 |
| *OLFM4* | 1.706 | 0.000 | 0.481 | 0.000 | 0.122 | 0.741 | 0.000 | -2.002 | -0.619 |
| *CRYAB* | 2.418 | 0.000 | 0.626 | 0.030 | 0.486 | 0.792 | 0.000 | -0.939 | -0.306 |
| *TFAP2B* | 1.613 | 0.000 | 0.576 | 0.001 | 0.466 | 0.727 | 0.002 | -0.449 | -0.261 |
| *KCNMA1* | 0.859 | 0.000 | 0.337 | 0.001 | 0.182 | 0.468 | 0.000 | -0.546 | -0.286 |
| *TFF1* | 1.561 | 0.000 | 0.267 | 0.001 | 0.149 | 0.372 | 0.009 | -0.602 | -0.223 |

Patient P8 cluster defining genes were selected compared to other CSF (cerebrospinal fluid) samples (***Figure 5I***) by fold-change FC > 1.5, pct in others ≤ 0.05 and adjusted *P*-value < 0.05; pct in P8: the percentage of cells where the gene is detected in P8 CSF samples; pct in others: the percentage of cells where the gene is detected in other CSF samples.
The 15 biased expression genes in P8-1 or P8-2 of P8 cluster defining genes were selected by pct in P8-1 or P8-2 < 0.5, |pct diff| > 0.2 and adjusted *P*-value < 0.05; pct in P8-1, the percentage of cells where the gene is detected in P8-1 CTCs; pct in P8-2, the percentage of cells where the gene is detected in P8-2 CTCs; pct diff, the different between the percentage of cells where the gene is detected in P8-1 CTCs and that in P8-2 CTCs (P8-1 minus P8-2).
P8 cluster defining genes with expression biased in P8-1 CTCs (9 genes) are shown in ***Figure 6D***, and with expression biased in P8-2 CTCs (6 genes) are shown in ***Figure 6E***.

**TABLE S7** List of specific genes up-regulated in LUAD-LM (lung adenocarcinoma leptomeningeal metastases) CSF-CTCs (cerebrospinal fluid circulating tumor cells) compared to normal CSF cells.

| Gene Name | $Log_2$ FC | *P*-adj | pct in CTCs | pct in Nor | pct diff | Freq | Gene Name | $Log_2$ FC | *P*-adj | pct in CTCs | pct in Nor | pct diff | Freq |
|---|---|---|---|---|---|---|---|---|---|---|---|---|---|
| *KRT19* | 4.536 | 0.000 | 0.939 | 0.243 | 0.696 | 5 | *SMIM22* | 2.035 | 0.000 | 0.704 | 0.034 | 0.670 | 5 |
| *IFI27* | 4.447 | 0.000 | 0.913 | 0.226 | 0.687 | 5 | *MET* | 1.982 | 0.000 | 0.606 | 0.034 | 0.572 | 4 |
| *KRT18* | 4.273 | 0.000 | 0.929 | 0.153 | 0.776 | 5 | *LGALS3BP* | 1.956 | 0.000 | 0.764 | 0.111 | 0.653 | 5 |
| *CEACAM6* | 3.987 | 0.000 | 0.893 | 0.217 | 0.676 | 5 | *PEG10* | 1.953 | 0.000 | 0.620 | 0.026 | 0.594 | 5 |
| *MMP7* | 3.739 | 0.000 | 0.643 | 0.064 | 0.579 | 4 | *TM4SF1* | 1.904 | 0.000 | 0.503 | 0.009 | 0.494 | 5 |
| *AGR2* | 3.669 | 0.000 | 0.864 | 0.136 | 0.728 | 5 | *KDELR2* | 1.897 | 0.000 | 0.716 | 0.106 | 0.610 | 5 |
| *RNASE1* | 3.580 | 0.000 | 0.850 | 0.115 | 0.735 | 5 | *TRIM24* | 1.896 | 0.000 | 0.606 | 0.132 | 0.474 | 5 |
| *NAPSA* | 3.448 | 0.000 | 0.935 | 0.060 | 0.875 | 5 | *HDLBP* | 1.875 | 0.000 | 0.841 | 0.200 | 0.641 | 5 |
| *KRT8* | 3.267 | 0.000 | 0.882 | 0.094 | 0.788 | 5 | *S100A13* | 1.859 | 0.000 | 0.570 | 0.030 | 0.540 | 5 |
| *MDK* | 3.259 | 0.000 | 0.794 | 0.111 | 0.683 | 5 | *CCND1* | 1.846 | 0.000 | 0.566 | 0.068 | 0.498 | 4 |
| *WFDC2* | 3.181 | 0.000 | 0.825 | 0.034 | 0.791 | 5 | *C3* | 1.836 | 0.000 | 0.589 | 0.149 | 0.440 | 4 |
| *SFTPB* | 3.121 | 0.000 | 0.917 | 0.149 | 0.768 | 5 | *IFI6* | 1.835 | 0.000 | 0.726 | 0.213 | 0.513 | 5 |
| *LMO7* | 2.897 | 0.000 | 0.842 | 0.068 | 0.774 | 5 | *SMIM1* | 1.832 | 0.000 | 0.404 | 0.004 | 0.400 | 4 |
| *CLDN7* | 2.827 | 0.000 | 0.812 | 0.081 | 0.731 | 5 | *CTTN* | 1.829 | 0.000 | 0.658 | 0.038 | 0.620 | 5 |
| *SCGB3A2* | 2.680 | 0.000 | 0.487 | 0.043 | 0.444 | 4 | *CAST* | 1.814 | 0.000 | 0.757 | 0.230 | 0.527 | 5 |
| *ADGRF1* | 2.658 | 0.000 | 0.710 | 0.051 | 0.659 | 4 | *TAGLN2* | 1.782 | 0.000 | 0.668 | 0.149 | 0.519 | 5 |
| *PIGR* | 2.574 | 0.000 | 0.459 | 0.043 | 0.416 | 4 | *CCT5* | 1.765 | 0.000 | 0.743 | 0.226 | 0.517 | 5 |
| *KRT7* | 2.573 | 0.000 | 0.767 | 0.060 | 0.707 | 5 | *C16orf89* | 1.756 | 0.000 | 0.525 | 0.034 | 0.491 | 4 |
| *SLPI* | 2.522 | 0.000 | 0.620 | 0.013 | 0.607 | 5 | *PLA2G16* | 1.721 | 0.000 | 0.667 | 0.128 | 0.539 | 4 |
| *APLP2* | 2.472 | 0.000 | 0.916 | 0.145 | 0.771 | 5 | *FOLR1* | 1.719 | 0.000 | 0.556 | 0.021 | 0.535 | 4 |
| *ERBB2* | 2.471 | 0.000 | 0.420 | 0.013 | 0.407 | 5 | *LGALS3* | 1.694 | 0.000 | 0.811 | 0.162 | 0.649 | 5 |
| *OSMR* | 2.457 | 0.000 | 0.720 | 0.085 | 0.635 | 5 | *MYO6* | 1.667 | 0.000 | 0.669 | 0.030 | 0.639 | 5 |
| *EGFR* | 2.442 | 0.000 | 0.587 | 0.030 | 0.557 | 5 | *PFDN2* | 1.656 | 0.000 | 0.768 | 0.149 | 0.619 | 5 |
| *CXCL17* | 2.426 | 0.000 | 0.786 | 0.055 | 0.731 | 5 | *NQO1* | 1.625 | 0.000 | 0.496 | 0.047 | 0.449 | 4 |
| *EMP2* | 2.405 | 0.000 | 0.705 | 0.051 | 0.654 | 5 | *LTO1* | 1.618 | 0.000 | 0.476 | 0.034 | 0.442 | 4 |
| *SLC34A2* | 2.403 | 0.000 | 0.826 | 0.043 | 0.783 | 5 | *YIF1A* | 1.612 | 0.000 | 0.742 | 0.102 | 0.640 | 5 |
| *MGST1* | 2.382 | 0.000 | 0.685 | 0.038 | 0.647 | 5 | *TARS* | 1.596 | 0.000 | 0.682 | 0.153 | 0.529 | 5 |
| *GDF15* | 2.345 | 0.000 | 0.574 | 0.055 | 0.519 | 5 | *STEAP4* | 1.594 | 0.000 | 0.467 | 0.021 | 0.446 | 4 |
| *SFTA2* | 2.320 | 0.000 | 0.673 | 0.000 | 0.673 | 5 | *PON2* | 1.593 | 0.000 | 0.617 | 0.017 | 0.600 | 5 |
| *CTSH* | 2.236 | 0.000 | 0.755 | 0.149 | 0.606 | 5 | *NT5DC2* | 1.590 | 0.000 | 0.606 | 0.064 | 0.542 | 5 |
| *RAI14* | 2.215 | 0.000 | 0.593 | 0.026 | 0.567 | 4 | *CD46* | 1.590 | 0.000 | 0.849 | 0.187 | 0.662 | 5 |
| *LMNA* | 2.201 | 0.000 | 0.816 | 0.077 | 0.739 | 5 | *CTNNA1* | 1.588 | 0.000 | 0.607 | 0.085 | 0.522 | 5 |
| *CKB* | 2.198 | 0.000 | 0.546 | 0.038 | 0.508 | 5 | *TXNDC17* | 1.587 | 0.000 | 0.745 | 0.115 | 0.630 | 5 |
| *MUC1* | 2.175 | 0.000 | 0.782 | 0.030 | 0.752 | 5 | *MAGED1* | 1.586 | 0.000 | 0.580 | 0.072 | 0.508 | 5 |
| *CDH1* | 2.154 | 0.000 | 0.625 | 0.047 | 0.578 | 5 | *DSTN* | 1.583 | 0.000 | 0.748 | 0.106 | 0.642 | 5 |
| *HSPD1* | 2.122 | 0.000 | 0.868 | 0.200 | 0.668 | 5 | *MGLL* | 1.552 | 0.000 | 0.547 | 0.030 | 0.517 | 5 |
| *ICAM1* | 2.105 | 0.000 | 0.590 | 0.157 | 0.433 | 5 | *EIF4G1* | 1.534 | 0.000 | 0.736 | 0.204 | 0.532 | 5 |
| *EPCAM* | 2.059 | 0.000 | 0.744 | 0.034 | 0.710 | 5 | *FLNB* | 1.521 | 0.000 | 0.476 | 0.043 | 0.433 | 5 |
| *ADGRF5* | 2.058 | 0.000 | 0.684 | 0.030 | 0.654 | 5 | *NAXE* | 1.505 | 0.000 | 0.700 | 0.094 | 0.606 | 5 |

Specific genes up-regulated in LUAD-LM CSF-CTCs compared to normal CSF cells were selected by $log_2$(fold-change) $log_2FC \geq 1$, pct in CTCs > 0.4, pct in Nor < 0.25, pct diff ≥ 0.4, adjusted *P*-value *P*-adj < 0.05, Frequent Freq ≥ 4; pct in CTCs, the percentage of cells where the gene is detected in the LUAD-LM CSF-CTCs group; pct in Nor, the percentage of cells where the gene is detected in the normal CSF cells group; pct diff: the difference between the percentage of cells where the gene is detected in CTCs and that in normal cells (CTCs minus Nor). Freq, a gene upregulated in the number of LUAD-LM patients.

*Supplementary Discussion*

**The transcriptome signature of CSF-CTCs in P8 CUP case.**

A total of 40 genes were selected as P8 cluster defining genes based on the gene expression profile (***Table S5 and Table S6***). In addition to *PIP* and *ANKRD30A*, some other genes also deserved our attention in spite of their unknown diagnostic value. *TFF1* (***Figure 6E***) and *TFF3* (***Figure 6B***) are members of the trefoil factor (TFF) family, which are typical constituents of mucous epithelium and commonly co-secreted with mucins (*MUC5AC* and *MUC5B*, **Figure. 6B and Table S5**).[1] *TFF1* and *TFF3* have been reported to interact with *MUC5AC* in gastric and ocular.[2] *MSMB* (microseminoprotein beta; ***Figure 6D***) is one of the most abundant proteins in semen[3] and has been used for diagnosis of prostate cancer[4]. *ZG16B/PAUF* (pancreatic adenocarcinoma-upregulated factor; ***Figure 6D***) is an important secreted protein in pancreatic ductal adenocarcinoma (PDAC) showing various crucial roles,[5] as well as in other cancer types including epithelial ovarian cancer,[6] cervical carcinoma,[7] colorectal cancer[8] and oral squamous cell carcinoma[9]. *KLK5* (Kallikrein-related peptidases 5; ***Figure 6D***), in the same gene family with the prostate-specific antigen *KLK3*, exerts key modulatory effects on many cancers and is selectively inhibited by SPINK, a family of serine peptidase inhibitor kazal type.[10] Interestingly, we discovered the higher expression of *SPINK8* (***Figure 6E***) in P8-2 CTCs compared to P8-1 CTCs, suggesting the potential interaction between *KLK5* and *SPINK8* during P8 disease progression. *OLFM4* (Olfactomedin-4; ***Figure 6E***) has important roles in various tumors and many cellular processes, especially in gastrointestinal malignancies with its primary function as an antiapoptotic factor.[11,12] Although the 40 P8 cluster defining genes provide useful information on the potential disease mechanism, they are still inconclusive in defining the CUP origin at the moment. As

more CSF scRNA-seq transcriptome are collected in the future, the value of these genes in the diagnosis of P8 will be developed to improve the diagnostic value and resolve the CUP origin.

**Supplementary References**


1. Braga Emidio N, Hoffmann W, Brierley SM, Muttenthaler M. Trefoil Factor Family: Unresolved Questions and Clinical Perspectives. *Trends Biochem Sci*. 2019;44:387-390.
2. Kjellev S. The trefoil factor family - small peptides with multiple functionalities. *Cell Mol Life Sci*. 2009;66:1350-1369.
3. Emami NC, Kachuri L, Meyers TJ, et al. Association of imputed prostate cancer transcriptome with disease risk reveals novel mechanisms. *Nat Commun*. 2019;10:3107.
4. Gronberg H, Adolfsson J, Aly M, et al. Prostate cancer screening in men aged 50-69 years (STHLM3): a prospective population-based diagnostic study. *Lancet Oncol*. 2015;16:1667-1676.
5. Kim SA, Lee Y, Jung DE, et al. Pancreatic adenocarcinoma up-regulated factor (PAUF), a novel up-regulated secretory protein in pancreatic ductal adenocarcinoma. *Cancer Sci*. 2009;100:828-836.
6. Choi CH, Kang TH, Song JS, et al. Elevated expression of pancreatic adenocarcinoma upregulated factor (PAUF) is associated with poor prognosis and chemoresistance in epithelial ovarian cancer. *Sci Rep*. 2018;8:12161.
7. Kim J, Chung JY, Kim TJ, et al. Genomic Network-Based Analysis Reveals Pancreatic Adenocarcinoma Up-Regulating Factor-Related Prognostic Markers in Cervical Carcinoma. *Front Oncol*. 2018;8:465.
8. Escudero-Paniagua B, Bartolome RA, Rodriguez S, et al. PAUF/ZG16B promotes colorectal cancer progression through alterations of the mitotic functions and the Wnt/beta-catenin pathway. *Carcinogenesis*. 2020;41:203-213.
9. Sasahira T, Kurihara M, Nishiguchi Y, et al. Pancreatic adenocarcinoma up-regulated factor has oncogenic functions in oral squamous cell carcinoma. *Histopathology*. 2017;70:539-548.
10. Kantyka T, Fischer J, Wu Z, et al. Inhibition of kallikrein-related peptidases by the serine protease inhibitor of Kazal-type 6. *Peptides*. 2011;32:1187-1192.
11. Wang XY, Chen SH, Zhang YN, Xu CF. Olfactomedin-4 in digestive diseases: A mini-review. *World J Gastroenterol*. 2018;24:1881-1887.
12. Zhang X, Huang Q, Yang Z, Li Y, Li CY. GW112, a novel antiapoptotic protein that promotes tumor growth. *Cancer Res*. 2004;64:2474-2481.
